# satmut_utils: a simulation and variant calling package for multiplexed assays of variant effect

**DOI:** 10.1186/s13059-023-02922-z

**Published:** 2023-04-20

**Authors:** Ian Hoskins, Song Sun, Atina Cote, Frederick P. Roth, Can Cenik

**Affiliations:** 1 Department of Molecular Biosciences, University of Texas at Austin, Austin, TX 78712 USA; 2 The Donnelly Centre and Departments of Molecular Genetics and Computer Science, University of Toronto, Toronto, ON Canada; 3 Lunenfeld-Tanenbaum Research Institute, Sinai Health, Toronto, ON Canada

**Keywords:** MAVE, DMS, Mutagenesis, Variant calling, SNP, MNP, *CBS*, Codon optimality

## Abstract

The impact of millions of individual genetic variants on molecular phenotypes in coding sequences remains unknown. Multiplexed assays of variant effect (MAVEs) are scalable methods to annotate relevant variants, but existing software lacks standardization, requires cumbersome configuration, and does not scale to large targets. We present satmut_utils as a flexible solution for simulation and variant quantification. We then benchmark MAVE software using simulated and real MAVE data. We finally determine mRNA abundance for thousands of cystathionine beta-synthase variants using two experimental methods. The satmut_utils package enables high-performance analysis of MAVEs and reveals the capability of variants to alter mRNA abundance.

**Supplementary Information:**

The online version contains supplementary material available at 10.1186/s13059-023-02922-z.

## Background

Multiplexed assays of variant effect (MAVEs) employ next-generation sequencing to profile the phenotypic effects of hundreds to thousands of genetic variants in a target gene. These assays, which rely on saturation mutagenesis, have been used to survey variant effects on molecular phenotypes ranging from mRNA and protein expression to protein binding and enzyme activity [1–10]. As a result, MAVEs emerged as methods to study variant effects and annotate variant significance, ultimately informing disease diagnosis and prognosis [11, 12]. Guidelines now exist for the development of MAVEs, underscoring their utility for variant annotation and interpretation [13]. Given saturation mutagenesis data contains variants with frequencies at and even below error rates for some polymerases (1 × 10^–4^), variant callers for MAVEs must have not only high sensitivity, but also high specificity. Yet, analysis methods for MAVEs are not standardized, and to our knowledge, none of the existing variant callers for analysis have previously benchmarked performance.

Existing tools for MAVE analysis require detailed configuration of parameters (“Methods”), may be limited to particular experimental designs, and fail to scale to large target genes. For example, dms_tools2 [14] has specific input requirements: primer designs should end flush with a codon, and reads must be dual-barcoded and align contiguously to a user-provided reference (no insertions or deletions). Similarly, Enrich2 [15] requires that reads align contiguously with a provided reference sequence. DiMSum [16] outputs nucleotide strings and only annotates amino acid changes in HGVS format. Importantly, all tools only call variants in a single PCR amplicon at a time. Hence, none of the published methods allow variant calling from multiple PCR amplicons at once with one configuration of analysis parameters, limiting the ability to rapidly scale to large genes. A recently released variant caller for MAVE analysis attempted to address the scalability problem, enabling both direct, “shotgun” sequencing and barcoded analysis [17]. However, this analysis did not benchmark performance against published MAVE variant callers.

Existing strategies typically assume pre- and post-selection sequencing of the variant library, for example when assaying variant effects on organismal growth [4]. While this is the predominant MAVE design, a generalized variant caller would facilitate not only selection-based assays but also assays of arbitrary design. Similarly, while a multitude of methods exist to call somatic variants in clinical samples [18–20], somatic variant callers for whole-transcriptome analysis are tailored to quantify variants in samples with few real single- and multi-nucleotide polymorphisms (SNPs and MNPs, respectively). In contrast, MAVE data contain a high density of thousands of low-frequency SNPs and MNPs. For example, di- and tri-nt MNPs may comprise a large proportion of the total variants in codon saturation mutagenesis. The low frequency of variants (< 1 × 10^−4^) pose new problems to variant calling for MAVE data. Analysis is further complicated by the hierarchical composition of variants, wherein true positive variants may be called together with nearby true or false positive variants [16].

To address the need to call low-frequency variants in MAVE data, we designed and implemented satmut_utils (saturation mutagenesis utilities), incorporating modern software development practices, extensive documentation, integration with package management, and rigorous unit testing. The satmut_utils “call” workflow is an end-to-end variant caller for MAVEs that supports direct analysis of targeted sequencing data from both (a) amplicon [4] and (b) rapid amplification of cDNA ends (RACE)-like library preparation methods [21–23]. Conversely, our software does not support analysis of barcoded-sequencing designs, or enable multi-codon (haplotype) variant calling, a requirement for analysis of experiments utilizing error-prone PCR (see Additional file 1 for rationale). To achieve high specificity, satmut_utils optionally builds on a simulation workflow (“sim”), enabling the generation of datasets for benchmarking and error modeling.

Here, we performed the first benchmarking analysis of MAVE variant callers on simulated and real MAVE data, and show that satmut_utils achieves superior performance for MAVE analysis. Using satmut_utils, we assayed variant effects on mRNA abundance using two library preparation methods. We identified variants in cystathionine beta-synthase (*CBS*) with effects on mRNA abundance, expanding a prior variant effect map for *CBS* function [10]. We further characterized possible mechanisms of altered mRNA abundance, including codon-mediated effects. The satmut_utils package enables flexible experimental design and comparative analysis of saturation mutagenesis data from various sources and will facilitate the interpretation of variant effects on multiple layers of gene expression.

## Results

### Design of simulation and variant calling workflows for saturation mutagenesis data

**Fig. 1:**
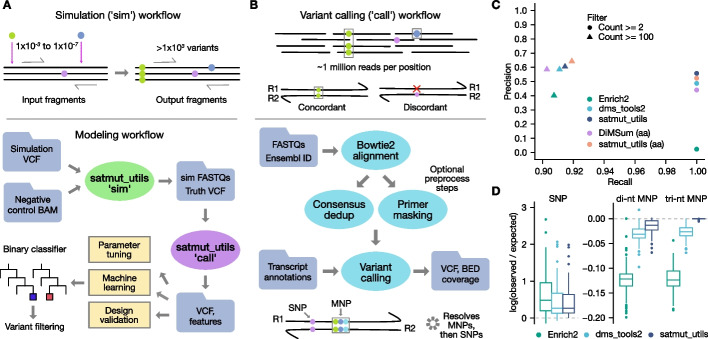
satmut_utils design and performance benchmarking. Solid circles represent single- or multiple-nucleotide polymorphisms (SNPs, MNPs), which may be either true or false positives (errors). **A** Variant simulation workflow. With “sim,” ultra-low-frequency variants in Variant Call Format (VCF) are edited into pre-existing sequencing read alignments (BAM). Edited reads (FASTQ) and true positive variants (Truth) are output with expected counts and frequencies. The “call” workflow **B** extracts quality features during variant calling, which may be used for assay design validation, software parameter tuning, and machine learning-based error correction. **B** Variant calling workflow. SNPs and MNPs are identified and quantified in paired-end reads following optional preprocessing to improve specificity. Transcript nucleotide and protein changes are annotated and a VCF and fragment coverage bedgraph file are output. **C** Performance of MAVE variant callers. Two hundred eighty-one variants were simulated in alignments for a single amplicon in *CBS*, and performance measures were evaluated after applying two simple count filters. nt: nucleotide/codon-level calls; aa: amino acid-level calls. **D** Accuracy of variant count estimates. The expected count is the simulated truth count. One outlier SNP was excluded for visualization (Enrich2 log ratio: 4.88; satmut_utils and dms_tools2 log ratio: 0.87). dedup = deduplication

We developed a workflow to simulate low-frequency variants in real alignments, termed “sim” (Fig. 1A). “sim” generates variants by editing into pre-existing alignments that correspond to a negative control (NC) sequencing library prepared from a non-mutagenized template. Editing real alignments enables us to capture sequencing errors and experiment-specific biases that may escape model-based in silico read generation. “sim” can efficiently simulate the number of variants typically targeted in MAVEs (> 1000) in a single transcript, improving on the scalability of existing solutions [24].

The “sim” workflow supports editing of multiple SNPs and MNPs at the same coordinate; ensures reads are edited only once; and allows the user to prohibit simulation of variants adjacent to pre-existing errors to ensure errors in the edited read do not convert the simulated variant to higher order (e.g., SNP to MNP; see Additional file 1). In summary, satmut_utils “sim” enables deterministic simulation of many low-frequency variants at the same position and offers the first generalized simulation method specific for multiplexed assays.

Next, to call low-frequency SNPs and MNPs in targeted sequencing data, we developed the satmut_utils “call” workflow (Fig. 1B). Importantly, “call” supports variant calling from multiple interleaved PCR tiles simultaneously, a feature lacking from other tools for analysis [14–16]. A curated human transcriptome is included to facilitate ease-of-use, although custom reference files are also supported. Our method provides two additional features missing in published MAVE analysis methods. First, satmut_utils enables variant calling from RACE-like library preparation methods such as Anchored Multiplex PCR [21] (Additional file 2: Fig. S1A). Second, satmut_utils extracts read-based quality data for each mismatch contributing to a variant call. Quality data may then be used to train error correction models. (For a detailed comparison of variant caller features see Additional file 2: Fig. S1B; for time and memory consumption of satmut_utils see Additional file 1).

To improve specificity of variant calls, the “call” algorithm incorporates filters based on read edit distance and base qualities (Additional file 2: Fig. S1C). Then, variants are called in read pairs if mates are concordant [4, 10], i.e., if the same base call is observed in both forward and reverse reads. This filters out sequencing errors which are found in only one read of the pair. Finally, satmut_utils employs a novel variant calling algorithm that prioritizes MNPs and improves sensitivity for MNP calls when they are adjacent to errors (Additional file 2: Fig. S1D). We coined the term *variant conversion* for cases when a true variant and adjacent error are called together as a false positive (Additional file 1). Conversion is particularly insidious for MAVE analyses as it may also lead to a false negative call. Altogether, satmut_utils (1) requires a single configuration for analysis of data from multiple amplicons; (2) supports two different library preparation methods; and (3) employs a unique variant calling algorithm for high-accuracy estimates of variant abundance.

### In silico validation and benchmarking of variant calls with “sim”

We compared performance of satmut_utils to dms_tools2 [14], Enrich2 [15], and DiMSum [16], in the first benchmarking analysis of MAVE variant callers. We speculate that a prior lack of benchmarking was due to several challenges: (1) lack of truth datasets; (2) different experimental design assumptions; and (3) non-standardized input and output file formats. Nonetheless, after preprocessing alignments to meet the various input requirements for Enrich2 and dms_tools2 (“Methods”), we successfully generated a common benchmarking dataset using reads from a single PCR amplicon in cystathionine beta-synthase (*CBS*) [10, 25]. This simulated dataset contained 281 variants at frequencies between 1 × 10^−6^ to 1 × 10^−3^ in a background of approximately two million negative control (wild-type) read pairs.

With a threshold of two supporting reads/fragments to make a variant call, Enrich2, dms_tools2, and satmut_utils achieved perfect sensitivity at the nucleotide level (Fig. 1C). However, precision was 0.023 (Enrich2, single-read mode), 0.487 (dms_tools2), and 0.553 (satmut_utils). Because DiMSum does not provide HGVS-formatted nucleotide annotations, but rather full nucleotide strings, we compared DiMSum to satmut_utils for amino acid changes (rationale in Additional file 1). At perfect sensitivity, DiMSum precision was 0.440 compared to satmut_utils precision of 0.605. Lower precision for Enrich2 and DiMSum may be due to merging with nearby errors. The satmut_utils “call” workflow does not call phased SNPs as a MNP unless the SNPs are within 3 nt (no haplotype calls are made). We found that this algorithmic design is a reasonable compromise to remove thousands of false positive calls arising from the merging of read errors. We note that Enrich2 precision might be higher with another analysis mode (barcoded sequencing; see Additional file 1 for a detailed explanation of benchmarking considerations).

Despite differences in overall performance, dms_tools2 and Enrich2 reported largely similar counts to satmut_utils for true positive variants, especially MNPs (Additional file 2: Fig. S2 A-C). Yet, satmut_utils reported more accurate variant counts than other methods for MNPs (Fig. 1D). Deviations from the truth count are likely impacted by read filtering and the variant calling algorithm (Additional file 2: Fig. S1 C-D), which may explain the higher accuracy of satmut_utils variant calling. In total, using satmut_utils “sim,” we performed the first benchmarking analysis of MAVE variant callers and showed that satmut_utils “call” is more accurate than other methods for variant calling of in silico mutagenesis data.

### “sim” and “call” power machine learning-based error correction

**Fig. 2:**
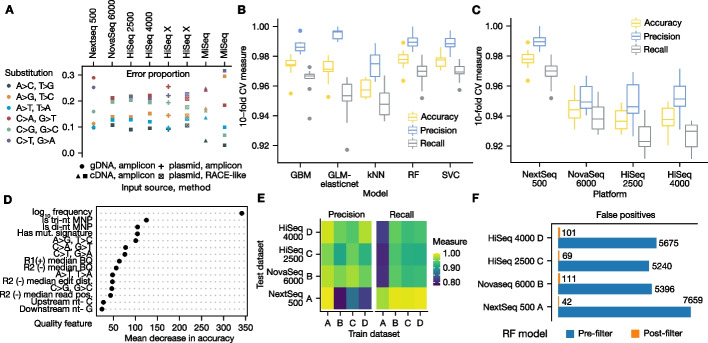
Machine learning models for error correction. Negative control (NC) alignments for “sim” dataset *A* (Nextseq 500) arose from the human *CBS* coding sequence after functional complementation in yeast [10]. Alignments for “sim” datasets *B–D* (NovaSeq 6000, HiSeq 2500, HiSeq 4000) and MiSeq runs arose from HEK293T endogenous *CBS* cDNA, and alignments for HiSeq X datasets arose from *CBS* plasmid. **A** Error proportions in negative control libraries. Proportion of each error substitution across NC libraries from various sources. Shape of the points indicates an independent NC library. **B** Model selection. To compare models, dataset *A* (3802 variants, 7859 true mismatches, 6463 false mismatches) was used. Up to 19 satmut_utils call quality features were selected to train binary classifiers (“Methods”). **C** Random forest performance. Random forests (RF) were trained on all four “sim” datasets and cross-validation performance across different platforms was calculated. **D** Feature importance for RF models. A RF was trained on a combined dataset (all “sim” datasets *A–D*), and the top fifteen important features as measured by mean decrease in accuracy (“Methods”) are plotted. **E** Cross-generalization of RF models. Pairwise train-test regimes were carried out with all “sim” datasets to assess model generalization across sequencing libraries and platforms. **F** Error correction impact on variant calls in NC libraries. satmut_utils variant calls from each NC library were filtered by the RF models. The number of error mismatches before and after filtering is plotted for each NC library. NC: negative control; GBM: gradient boosted machine; GLM-elasticnet: generalized linear model with elastic net regularization; kNN: *k*-nearest neighbors; RF: random forest; SVC: support vector classifier

Sequencing libraries contain systematic errors arising from library preparation- and sequencer-specific biases [26–28]. In MAVEs, a negative control (NC) library of the non-mutagenized template is typically sequenced in the same experiment as mutagenized libraries [10]. In agreement with prior observations of experiment- and platform-specific errors [28], we found a wide range of error rates for independent libraries from various labs, experiments, and sequencing runs (Fig. 2A). We noted the highest error rates for (C > A, G > T) and (C > T, G > A) substitutions across all Illumina platforms, library preparation methods, and independent libraries from various input nucleic acid sources. We hypothesized that sequencing a NC library, simulating variants in this control, and then training classifiers would help moderate such biases.

To test the utility of error correction models enabled by satmut_utils, we generated four large simulated datasets by editing thousands of variants into two NC libraries, sequenced on four Illumina platforms (“Methods”). At perfect recall, satmut_utils precision in these datasets with default calling parameters and no model-based error correction (“Methods”) was 0.552 + / − 0.041 (mean + / s.d.). Thus, with a naïve filter using a minimum count threshold, thousands of false positives remain in multiplexed assay datasets, deteriorating their quality.

We next used the simulated dataset from the first NC library, which comprised the human *CBS* coding sequence after functional complementation in yeast [10], to assess performance of machine learning models in reducing false positives. We trained binary classifiers using quality features extracted by satmut_utils “call” from the first simulated dataset (hereafter dataset *A*). Of the five classifiers evaluated by cross-validation, all five models showed a median accuracy > 0.95 (Fig. 2B). The remaining three datasets (*B–D*) arose from a second NC library consisting of the *CBS* coding sequence amplified from human HEK293T total RNA and sequenced on different platforms. We selected the random forest (RF) to test performance on all four datasets generated with “sim” (Fig. 2C). The mean accuracy of the final models (*N* = 4) on an independent test set was 0.954 + / − 0.019 (mean, s.d.), indicating that models trained on simulated data are robust to different choices of NC library and sequencing platform, and outperform filtering variants using a fixed count threshold (Fig. 1D). Several quality features lent predictive power as measured by RF feature importance (Fig. 2D, Additional file 3: Table S1).

To assess generalization of the models, we trained a RF on one simulated dataset and tested it on all other datasets (all pairwise permutations, Fig. 2E). Models generalized well for our own NC library sequenced on different platforms, with an accuracy of 0.938 + / − 0.014 (mean, s.d.). Accuracy was slightly worse when trained on the independent dataset *A* and tested on datasets *B-D*: 0.891 + / − 0.012 (mean, s.d.). We finally applied the models to filter calls in the NC libraries and observed a strong reduction of false positives (Fig. 2F). Therefore, training error correction models on simulated data appreciably improves variant calling precision (see Additional file 1 for potential caveats).

### Read preprocessing implemented in satmut_utils reduces false positive variant calls

**Fig. 3:**
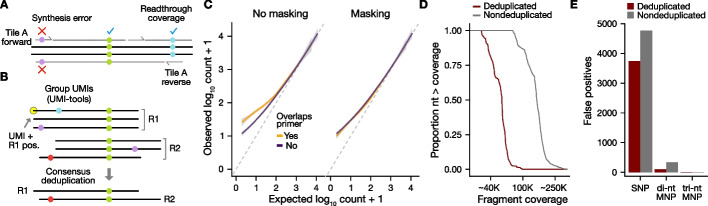
Read preprocessing for error correction. For **A** and **B**, solid colored circles represent SNPs, either true or false positive (error). **A** Primer base quality masking schematic. Base qualities for read segments determined to originate from synthetic primer sequences are set to 0. Black lines indicate the sequenced fragment. Solid gray lines with ticks represent primers/directionality. Gray dotted lines represent reads off the input fragment. Readthrough coverage refers to coverage from adjacent PCR tiles, required to call variants that overlap primers. **B** Consensus deduplication schematic. UMI-tools directional adjacency method [75] is used to group paired-end reads from a common unique fragment, defined by UMI and read 1 position (R1 pos.). A custom consensus deduplication algorithm generates the consensus base among duplicates at each aligned fragment position for each read. **C** Primer base quality masking improves accuracy of variants underlying primers. Simulated datasets (Fig. 2, *N* = 4) were analyzed with/without primer BQ masking and true positive variants that overlap primers are plotted compared to variants not overlapping a primer. **D** Consensus deduplication maintains coverage uniformity. A UMI-containing, RACE-like negative control (NC) library was generated. Waterfall plots of cumulative fragment coverage for consensus-deduplicated reads and non-deduplicated reads indicate uniform collapse of PCR duplicates. *x*-axis is in log_10_ scale with a range between 4.6 and 5.6. **E** Consensus deduplication reduces false positives. The effect of consensus deduplication is shown for the RACE-like NC library for each variant type. UMI = Unique molecular index; SNP = single-nucleotide polymorphism; MNP = multiple-nucleotide polymorphism; NC: negative control

While machine learning models using sequence-level features reduce false positives, additional improvements that leverage read preprocessing can further improve specificity. For example, primer base quality masking [20] may be used to omit variant calls that arise from primer synthesis errors, by setting base qualities to 0 for synthetic read segments (Fig. 3A). When unique molecular indices (UMIs) are incorporated into the library design, further improvements can be obtained by consensus deduplication [14, 20, 29], where a consensus sequence is generated from PCR read duplicates (Fig. 3B). We implemented these additional methods and compared variant calls in simulated datasets before and after primer masking and consensus deduplication using UMIs.

Primer masking removed a small number of false positive SNPs in “sim” datasets (min = 1, max = 64; min proportion of SNPs remaining = 0.985). More importantly, primer base quality masking improved the accuracy of variant counts in simulated data (Fig. 3C, N = 4 pooled datasets). In parallel with primer masking, consensus deduplication of a RACE-like (Anchored Multiplex PCR) NC library through UMIs reduced depth of coverage across *CBS* by 63.1% (Fig. 3D, “Methods”). Further, deduplication reduced false positive (FP) SNPs by 21.5% (1026 FPs); di-nt MNPs by 70.3% (237 FPs); and tri-nt MNPs by 27.2% (3 FPs) (Fig. 3E). This significant improvement in specificity may be accompanied by a slight cost to sensitivity, but the current implementation of the “sim” workflow was insufficient to determine the exact sensitivity–specificity tradeoff (see Additional file 1 for details). Altogether, read preprocessing steps can improve the quality of MAVE data prior to variant calling, independent of other model-based error correction.

### End-to-end analysis of growth-based MAVE data with satmut_utils

To apply satmut_utils to real MAVE data and compare the impact of variant calling algorithms on fitness estimates, we used two growth-based MAVE experiments- human *SUMO1* [4] and influenza *HA* [30]. We quantified variant counts using satmut_utils “call” or DiMSum “WRAP,” then applied a uniform statistical inference pipeline to determine variant fitness effects (DiMSum “STEAM,” “Methods”). This approach enabled us to tease-apart the impact of variant calling algorithms on the resulting fitness estimates and variant effect maps.

**Fig. 4:**
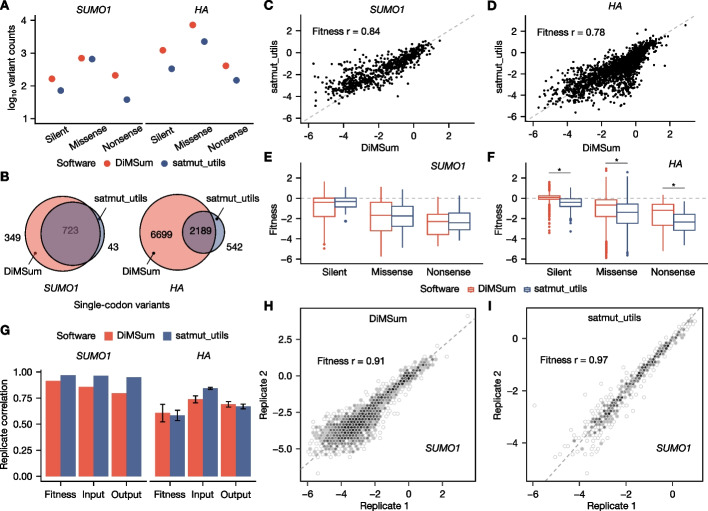
End-to-end analysis of growth-based MAVE datasets with satmut_utils and DiMSum. *SUMO1*: *Homo sapiens* small ubiquitin-like modifier 1. *HA*: influenza A/WSN/1933(H1N1) hemagglutinin. In all panels, all six amplicons in the *HA* dataset [30] were analyzed, and only one of the three targeted amplicons in the *SUMO1* dataset [4] was analyzed for simplicity (Methods). For **A–F**, only “single” variants are plotted (those altering only one codon/amino acid). For **C**, **D**, **H**, and **I**, the Pearson correlation coefficient for DiMSum fitness estimates is indicated. In **E** and **F**, outliers are greater than 1.5 * interquartile range. **A** Variant counts by software method. A pseudocount of 1 was added. **B** Variant call overlap by software. **C** *SUMO1*, and **D** ***HA*** fitness estimates by software. **E** *SUMO1*, and **F** *HA* fitness estimate distributions by variant type. Asterisks indicate significant two-sided Wilcoxon rank-sum tests at a threshold of < 1 × 10^−10^.** G** Biological replicate correlation. Shown is correlation between fitness estimates, input counts, and output counts. The *SUMO1* dataset contained two replicates. For *HA* dataset triplicates, the mean correlation + / − the standard deviation is shown. **H** DiMSum, and **I** satmut_utils replicate correlation for *SUMO1*. Scatterplots derive from DiMSum reports which aggregate counts for visualization

We found that DiMSum reported more variants than satmut_utils for both datasets (Fig. 4A,B). This is partly due to satmut_utils’ leverage of the NC library for error correction (i.e., subtraction of background errors, “Methods”). Nonetheless, satmut_utils and DiMSum reported well-correlated fitness estimates for both datasets (Fig. 4C,D; Pearson correlation 0.84 and 0.78 for *SUMO1* and *HA*, respectively). We hypothesized that more accurate software should lower the fitness estimates for missense and nonsense variants relative to silent variants, so we compared fitness estimates between these variant types. For the *HA* dataset, but not the *SUMO1* dataset, we found satmut_utils reported lower fitness estimates for missense and nonsense variants compared to DiMSum (Fig. 4E,F). Conversely, the biological replicate correlation of raw counts and fitness scores was higher for satmut_utils in the *SUMO1* dataset (Fig. 4G–I). Our analysis indicates satmut_utils accurately quantifies variants in large target regions with multiple amplicons.

Altogether, we found variant calling algorithms and pre-/postprocessing methods have a measurable impact on fitness estimates. Importantly, our analysis demonstrates the modularity and scalability of satmut_utils, and its integration with secondary analysis modules to compute fitness estimates.

### Variant calls are reproducible by two orthogonal library preparation methods

**Fig. 5:**
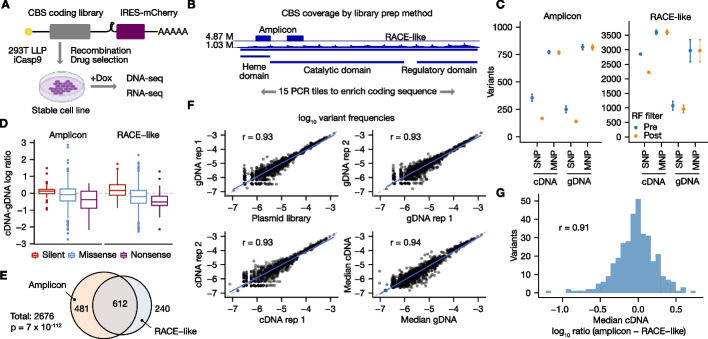
satmut_utils analysis of a *CBS* variant library by orthogonal library preparation methods. **A** Experimental strategy. A human *CBS* coding variant library was stably expressed in a landing pad cell line [31]. gDNA and total RNA were sequenced by one of two targeted library preparation methods 24 h after induction with Doxycycline (Dox). **B** *CBS* domains and sequencing coverage. Coverage for two tiles by the amplicon method is contrasted with full coverage in the RACE-like method. The maximum coverage depth is shown on the left of the track. **C** Filtering of variant calls. Random forest models were trained for each method using negative control libraries and the “sim” workflow. Plotted are the mean number of variant calls + / − the standard deviation (*N* = 3). The total possible calls are as follows: amplicon (531 SNPs, 2145 MNPs); RACE-like (4105 SNPs, 16,863 MNPs). **D** Differential abundance for mutation types. The difference in the median log frequency between cDNA and gDNA is shown for variants observed in all gDNA and cDNA replicates (*N* = 6). Outliers are greater than 1.5 * interquartile range. Asterisks indicate significant differences by one-sided Wilcoxon rank-sum tests (*p* < 0.05). **E** Variant call overlap between methods. Overlap in variant calls is shown for *CBS* tiles 2 and 4. Total calls are the theoretical number of variant calls. Significance of overlap was computed by a hypergeometric test. **F** Biological replicate correlation for amplicon libraries. Reproducibility between log_10_ frequencies was determined by Pearson’s correlation coefficient after filtering (“Methods”). **G** Similarity of variant frequency estimates between methods. The difference in median log_10_ frequency between methods is shown for cDNA libraries with filtering and correlation as in **F**. M = million

To date, most MAVE studies have measured variant effects on protein fitness or organismal fitness. We sought to show the flexibility of satmut_utils in analyzing datasets that diverge from the common growth-assay design. To this end, we measured gDNA and mRNA abundance for a complete coding variant library in *CBS*, following stable expression in a HEK293T landing pad cell line [31]. Our design enabled the measurement of variant effects on gene expression at the nucleotide level. We recombined a *CBS* variant library [10] into the landing pad line with a downstream IRES-mCherry element (Fig. 5A). Then, we assayed variant abundance in gDNA and cDNA by amplicon [4, 10] and RACE-like (Anchored Multiplex PCR) [21] library preparation methods (“Methods”). The quality of total RNA as well as PCR products at steps in library preparation was confirmed (Additional file 2: Fig. S3).

For each method, we again included a NC sequencing library to enable variant filtering with a random forest (RF) model. These libraries showed uniform coverage across the *CBS* target regions (Fig. 5B), as did mutagenized libraries. We found high performance of RF models for both library preparation methods (0.975, 0.959 accuracy for amplicon and RACE-like simulated datasets, respectively). These models were subsequently used to filter variant calls from the mutagenized sequencing libraries (Fig. 5C).

The difference in log ratios (variant:wild-type) between cDNA and gDNA for each variant highlighted large effects on relative abundance in both directions. As expected, missense and nonsense variants reduced mRNA abundance compared to silent changes (Fig. 5D). Variant calls made by both library preparation methods comprised 22.9% of the maximum theoretical calls for the two amplicons (2676), and overlap was significant by a hypergeometric test (Fig. 5E, p = 7 × 10^–112^). For the amplicon method, among variants detected at least once, 55.7% of these variants were found in all replicate gDNA and cDNA libraries (*N* = 3 replicates each). In contrast, only 15.9% of variants were observed in all replicates by the RACE-like method.

Variant abundance estimates were reproducible across input sources and independent biological replicate cell lines (Fig. 5F, Additional file 2: Fig. S4 A-B). Despite a difference in coverage depth (Fig. 5B), the variant frequency correlation was satisfactory compared to the RACE-like method for variants that were well-measured (0.91 Pearson’s correlation, Fig. 5G, Additional file 2: Fig. S4C). Taken together, our results suggest satmut_utils reports reproducible variant frequency estimates from two library preparation strategies and facilitates analyses from multiple nucleic acid sources.

### Identification of CBS variants that alter mRNA abundance

To apply satmut_utils variant calling to unveil biological insights, we next determined *CBS* variants with effects on mRNA abundance. The human CBS enzyme has specific amino acids that bind two cofactors (heme and pyridoxal phosphate (PLP)) [32–34]. These cofactors regulate folding, stability, and activity of CBS [10, 35–37]. Because heme and PLP can stabilize CBS variants and remediate pathogenic phenotypes [10, 38, 39], and because heme binding is not reversible [40], we hypothesized heme facilitates co-translational folding of CBS, similar to its role in folding of globin [41, 42].

**Fig. 6:**
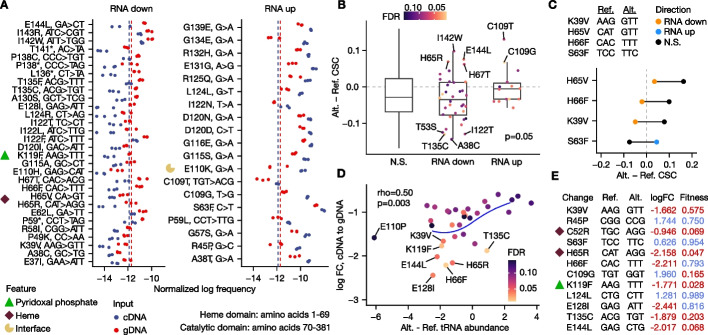
Identification and mechanisms of CBS variants that alter mRNA abundance. In all panels, data from the amplicon method is shown, and a gray dotted line indicates no change in variant effect or comparative metrics. For panel **A**, variants at a FDR < 0.1 are shown; in all other panels, variants with a FDR < 0.15 were analyzed. **A** CBS variant differential abundance. Structural residues near important features are labeled with an icon. Red and blue dotted lines represent the median for each input source. **B** Codon stability coefficient for variants grouped by directional effect. The difference in HEK293T ORFome codon stability coefficient (CSC) scores [52] between alternate and reference codons is compared. *p*-value indicates a one-sided Wilcoxon rank-sum test between RNA down and RNA up groups. **C** Comparison of codon stability between significant and non-significant variants. Significant variants were compared to another variant leading to the same amino acid change. Variants in the upper quartile of a null distribution are shown (“Methods”). **D** tRNA abundance correlation with mRNA abundance effects. The log fold change (logFC) is compared to the log ratio of tRNA abundance measured by Hydro-tRNAseq [56]. Spearman rank correlation (rho) and *p*-value are shown. Blue line is a fitted polynomial spline with a knot at 0. **E** Comparison of mRNA abundance effect with yeast functional complementation. Score (range 0–1, 0 is deleterious) is the max score of low and high vitamin B6 conditions [10]. Red text indicates a fitness score < 0.7 or a logFC < 0. Ref. = reference codon; Alt. = alternate codon; N.S. = not significant

We reasoned that *CBS* variants with low mRNA abundance may be enriched at or near important structural residues of the CBS protein, as improper co-translational folding may trigger ribosome quality control, leading to mRNA and protein degradation [43, 44]. To address this hypothesis, we determined *CBS* variants with significant differential abundance between cDNA (total RNA) and gDNA using the high-quality data from the amplicon method (Fig. 6A, Additional file 2: Fig. S5A, “Methods”). Of the 2676 theoretical variants for the amplicon method, 1240 were detected (46.3%) at least once, and 691 were observed in all gDNA and cDNA replicates (*N* = 6, 25.8%). Of these, 19 variants were higher in mRNA abundance compared to 30 variants that were lower in mRNA abundance (FDR < 0.1).

Several variants at and near important residues for activity, including variants at positions previously implicated in CBS deficiency, exhibited significant effects on mRNA abundance (Additional file 3: Table S2). Specifically, we identified decreased mRNA abundance of the variant K119F, which interrupts the Schiff base formed by this residue with PLP [32]. Similarly, mutations at or adjacent to the heme-binding residue (H65) exhibited a strong reduction in mRNA abundance: H65R, H65V, H66F, and H67T. Variants with decreased mRNA at positions previously implicated in CBS deficiency included P49K, R58I, E128I, I143R, and E144L [37, 45–48].

Fifty other variants had differential mRNA abundance by the RACE-like method (Additional file 2: Fig. S6A, FDR < 0.1), and despite limited variants being detected by both preparation methods in all replicates, we found a high correlation in the mRNA abundance effect between amplicon and RACE-like methods (0.998 Pearson’s coefficient, Additional file 2: Fig. S6B). We noted variant effects that depend on the position of the variant in the coding sequence. In the amplicon method, the variance of the effect at each position was higher in the catalytic domain than in the heme domain, suggesting the magnitude of *CBS* variant effects may depend on the encompassing structure of the CBS protein (Levene’s test, *p* = 0.009, Additional file 2: Fig. S5B). Similarly, RACE-like data indicated nonsense variants had the strongest effects on mRNA abundance when located near the middle (catalytic domain) of the coding sequence (Additional file 2: Fig. S6C).

Altogether, we identified *CBS* variants near important functional residues that alter mRNA abundance. Consistent with co-translational folding of CBS by cofactors, mutations at and adjacent to the heme- and PLP-binding residues exhibited decreased mRNA abundance. Other mutations in these domains showed increased mRNA abundance, suggesting complex regulation of *CBS* mRNA expression linked to other nucleotide or codon features.

### Differential mRNA abundance is consistent with codon-mediated stability and identifies variant effects undetected at the protein level

In yeast, zebrafish, *Xenopus*, and human cells, mRNA decay and translation efficiency are partially explained by codon optimality [49–54], where optimal codons are defined as those enriched in transcripts with longer mRNA half-lives and/or increased translation efficiency. While previous studies predominantly relied on reporter assays to assess the impact of codon optimality on these gene expression phenotypes, our approach enabled testing the relationship in a native coding sequence. To test if any *CBS* variants alter mRNA abundance through codon-mediated mRNA stability, we compared the difference in tRNA abundance and the codon stability coefficient (CSC) [52, 55] between alternate and reference codons to the magnitude of variant effect (“Methods”).

The difference in CSC [52] between alternate and reference codons was lower for variants with decreased mRNA abundance compared to variants with increased abundance, indicating changes to less-stable codons may reduce mRNA levels (Fig. 6B, one-sided Wilcoxon rank-sum test *p* = 0.049). Notably, mutations to cysteine (A38C, T135C, P138C, N327C, A509C) and from cysteine (C109G/T) exhibited effects consistent with low stability of the UGU codon [52] (Additional file 3: Table S2). By comparing each differential variant to a non-significant variant leading to the same amino acid change, we found 13 variants had a CSC difference in the expected direction compared to 5 variants in the unexpected direction (binomial test *p* = 0.02). We highlighted the top four variants with CSC differences in the expected direction in Fig. 6C. Similarly, tRNA abundance exhibited a modest correlation with the mRNA abundance effect for variants down in mRNA (0.50 and 0.28 Spearman correlation for Hydro-tRNAseq [56] and mim-tRNAseq [55], respectively) (Fig. 6D, Additional file 2: Fig. S5C). We propose changes to the cysteine codon UGU, valine codon GUU, and phenylalanine codon UUU may reduce mRNA abundance (Additional file 3: Table S2). Our results suggest codon optimality partially explains mRNA abundance effects for at least some missense variants.

Finally, we integrated paired yeast functional complementation data for the *CBS* variant library [10] and found 89/191 (46.6%) of variants called by both methods at a FDR < 0.15 showed a consistent directional effect (e.g., low mRNA, low fitness) at a fitness score cutoff of 0.7 (score range 0–1; 0 is deleterious; binomial test *p* = 0.81). This indicates no significant global agreement between assays in human and yeast; however, variants with the most significant negative effects were deleterious by functional complementation (Fig. 6E). 18.5% of missense variants (35/189) with low mRNA abundance were at amino acid positions implicated in CBS deficiency. Of variants with decreased mRNA abundance, low fitness was observed for H65R/V, E110H, K119F, D120I, I122F/L/T, L124R, T135C, I142W, I143R, E144L; other variants showed a more modest reduction of fitness (E37I, A38C, K39V, P49K, R58I, E62L, H66F, H67T, G115A, E128I, A130S, T135F, P138C). Our results highlight the utility of mRNA abundance readouts to complement organismal fitness data. Together, we find variant effects on mRNA abundance are partially explained by codon-mediated stability and may diverge from yeast functional complementation readouts.

## Discussion

The explosion of saturation mutagenesis studies [2, 5–7, 9, 10, 57–59], facilitated by next-generation tools to measure molecular phenotypes [4, 5, 31, 60], prompts a need for an analysis solution that is extensible to multiple experimental designs. We created satmut_utils to fill this gap by providing simulation and variant calling in whole coding sequences for both amplicon and RACE-like library preparation methods.

With satmut_utils “sim,” we conducted the first benchmarking analysis of MAVE variant callers, and trained error correction models to achieve high variant calling performance. We further implemented several read preprocessing strategies (primer masking, consensus deduplication), which act synergistically with error correction models to improve specificity. Our goal with satmut_utils “call” was to enable primary variant calling analysis to accurately resolve low-frequency SNPs and MNPs. The previously developed software methods for analysis of MAVEs [14–16] do not easily scale for large genes and are tailored to pre-/post-selection designs. We developed a general solution that makes limited assumptions about experimental design and focuses on accurately identifying and quantifying variants prior to statistical inference.

One limitation of satmut_utils is that it is not compatible with barcode-sequencing, wherein a barcode (i.e., randomer) is separated from the mutagenesis region and linked to a specific variant. While this method simplifies variant quantification, it requires initial sequencing for barcode assignment, which increases cost and time. Further, compared to direct variant calling, barcode-sequencing may lead to regulatory changes due to molecular linking between the barcode and the gene of interest. In addition, satmut_utils does not call phased variants that lead to multi-codon/amino acid variants, which may be generated by error-prone PCR. This design choice was informed by simulation and reduces *variant conversion* (Additional file 1).

We demonstrate the utility of our software solution by re-analyzing two growth-assay datasets, highlighting satmut_utils’ flexibility in processing data from various experimental designs. Then, building on prior clinical and functional data [10], we assayed variant effects on *CBS* mRNA abundance in human cells and found several variants at important CBS structural residues with low mRNA abundance. Our results are consistent with a recent study that employed saturation genome editing of *BRCA1* to uncover hundreds of SNPs with differential mRNA abundance. This study also supported the notion that variants at key structural residues can lead to low mRNA abundance [61].

Both synonymous and nonsynonymous variants may have strong effects on expression through translation regulation, codon optimality, and alteration of mRNA secondary structure [62–68]. Optimal codons tend to be enriched in regions encoding buried, non-solvent accessible residues [69], which may explain our observation of a dependence of the magnitude of variant effect on position in the coding sequence. We also identified several variants that converted to a non-optimal cysteine codon (UGU) and exhibited low mRNA abundance, consistent with its low codon stability [52]. We speculate mutations causing CBS misfolding may negatively feed-back on *CBS* mRNA abundance via reduced biosynthesis of cysteine, compounding the deleterious effect of low stability of the UGU codon. Further work is needed to quantify the extent to which codon optimality modulates expression of endogenous transcripts [61] as opposed to reporter constructs, and satmut_utils is poised to support such studies.

While mRNA abundance effects are consistent with codon stability and tRNA abundance data, there was no global relationship with yeast functional complementation data. This may be due to known differences in regulation of human and yeast CBS (requirement for heme, S-adenosylmethionine) [32, 35, 70]. However, variants with the largest effects in our study primarily agreed with readouts in yeast.

Here, we analyzed coding variant effects on mRNA abundance in human cells, and effects from prior yeast functional complementation or viral fitness datasets [4, 30], but analysis of other MAVE data is possible. For example, FACS-based assays to measure protein abundance [5, 7, 9] are plausible applications for satmut_utils analysis.

## Conclusions

We offer satmut_utils as a flexible solution for variant simulation and variant calling in saturation mutagenesis experiments. The satmut_utils package is unit-tested, well-documented, and available on GitHub. Our method is flexible to design, supports two different library preparation methods, and incorporates state-of-the-art error correction by read preprocessing and machine learning models. Further, satmut_utils uses standardized input and output files and is compatible with existing statistical inference tools. In conclusion, satmut_utils is a complete solution for multiplexed assay variant calling and will motivate novel assays based on targeted DNA and RNA sequencing.

## Methods

### satmut_utils “sim” workflow

The satmut_utils “sim” workflow takes a Variant Call Format (VCF) and alignment (BAM) file with paired reads as input and generates variants in the reads at specified frequencies. Outputs are a VCF of true positive (truth) variants and counts, along with edited reads (FASTQ). “sim” is comprised of three overall steps: (1) a single samtools “mpileup” call [71, 72] is made to query reads at each position in the target region. The number of fragments to edit and the read positions to edit are determined for each variant based on specified frequencies in the input VCF. “sim” employs a heuristic to sample reads for editing at each target position while prohibiting variant conversion (the merging of edited variants with nearby errors). (2) With these edit configurations, variants are edited into read pairs and written as raw reads in FASTQ format by “samtools fastq.” (3) The raw reads are re-aligned with bowtie2 [73] *global* alignment mode to generate valid CIGAR and MD tags, which are required for visualization of edited reads in genome browsers.

### satmut_utils “call” workflow

satmut_utils “call” utilizes cutadapt [74] for adapter and 3′ base quality trimming, followed by an optimized, paired-end *local* alignment to the transcript reference using bowtie2 [73] with the following parameters: “–maxins = 1000 –no-discordant –fr –mp 4 –rdg 6,4 –rfg 6,4.” If consensus deduplication is requested, this step directly follows alignment. Then, if a primer BED file is provided, primer base quality masking is performed. Following read preprocessing, filtering on base quality, read edit distance, and min supporting counts is applied during variant calling. Variant calls are made by iterating over filtered read pairs, finding mismatches with mate concordance, extracting quality features, and *writing results for each mismatch participating in a primary variant call*. (This should be considered when counting records from output files). Fragment coverage depth is reported in bedgraph format. To validate satmut_utils “call,” we generated in silico, error-free, paired-end RNA reads and then introduced 187 SNPs and MNPs, each at 10 read pairs in 10,000 (0.1%). Tuning of bowtie2 InDel penalties was required to achieve 100% recall for MNPs (Additional file 2: Fig. S1E).

### satmut_utils primer base quality masking

If a primer BED file is provided, alignments are intersected with primers with “bedtools intersect -bed -wa -wb.” The resulting BED file is processed with “bedtools groupby -o collapse” to group the intersecting primers for each read, and primers which originate the read are determined by the following criteria: (1) the read 5′ end begins within the aligned coordinates of the primer, or starts within a buffer upstream of the primer 5′ end (relative to strand); (2) the read 3′ end stops within the aligned coordinates of a primer on the opposite strand, or stops within a buffer upstream of the primer 5′ end (relative to strand). The buffer is 15 nt for amplicon methods and 3 nt for RACE-like methods. (These rules ensure masking for 3′ base quality trimmed reads and reads with slight differences in alignment start and stop coordinates, for example due to incomplete primer synthesis or alignment clipping). Subsequences for originating primers are masked in the reads by setting the base qualities of these read segments to 0. These bases are not subsequently considered for variant calling and fragment coverage enumeration.

### satmut_utils UMI-based consensus deduplication

Unique molecular indexes (UMIs) are extracted to the read header prior to adapter and 3′ base quality trimming. Following alignment, reads are grouped by [UMI × R1 POS] with UMI-tools [75] default directional adjacency method and the “–paired, –ignore-tlen” flags. Group ID tags are copied from R1 alignments to R2 alignments, paired reads are combined and sorted by read name, and then R1 and R2 are consensus deduplicated using a majority vote at each aligned position in the UMI group. In base call ties (two duplicates), if one base call matches the reference base, the reference base is used for the consensus. Otherwise, the base call with the higher base quality is used, thereafter defaulting to random choice.

### Data preprocessing for benchmarking

Reads originating from a single *CBS* negative control amplicon (tile 6) from the wild-type, non-selected condition [10] were selected for simulation. To meet dms_tools2 and Enrich2 input requirements, reads were preprocessed using the DesignConverter class in v1.0.1-dev001 [76]. A script (run_design_conversion.py) is now provided in satmut_utils v1.0.3-dev001. Preprocessing comprised several steps: (1) reads were locally aligned; (2) any hard- or soft-clipped reads, unpaired singletons, and reads with InDels were filtered out; (3) reads were modified to start and end flush with codons by trimming and/or appending reference sequence; (4) for dms_tools2, 12 nt unique molecular indices (UMIs) were added to the 5′ end of both R1 and R2, enforcing unique UMIs for each read pair.

An accessory script (run_ec_data_generator.py) [76] was used to generate the benchmarking dataset. Two hundred eighty-one SNPs and MNPs were simulated in the preprocessed negative control (NC) alignments for tile 6 of *CBS*, using frequency parameters estimated from satmut_utils variant calls (-m 2 -q 30 -e 10 -s NNK) across all tiles of the mutagenized, non-selected condition [10]. In addition, the proportion of SNPs in the truth set was set at 0.25. To balance the number of true and false positive labels, the number of variants to edit was determined by a heuristic that samples variants until the number of component mismatches comprising these variants equals the number of false positive mismatches in the NC library. The number of false positive mismatches in the NC library was determined by satmut_utils call using the following parameters: “-m 1 -q 30 -e 10 -s NNK.”

### Benchmarking analysis

Configurations and quality parameters for benchmarking were as follows.

CBS_TILE6_SEQ = “GACGTGCTGCGGGCACTGGGGGCTGAGATTGTGAGGACGCCCACCAATGCCAGGTTCGACTCCCCGGAGTCACACGTGGGGGTGGCCTGGCGGCTGAAGAACGAAATCCCCAATTCTCACATCCTAGACCAGTACCGCAACGCCAGCAACCCC”.

1. DiMSum version 1.2.9: “–stranded = T -q 30 -m 10 -u coding –mutagenesisType = codon –indels = none –mixedSubstitutions = T -w $CBS_TILE6_SEQ” Variants with one count were filtered out.
2. dms_tools2 version 2.6.10: “–alignspecs 19,132,31,34 –bclen 12 –bclen2 12 –chartype codon –maxmuts 10 –minq 30 –minreads 1”
  Variants with one count were filtered out.
3. Enrich2 version 1.3.1: {"filters": {"avg quality": 20, "max N": 10}; "variants": {"max mutations": 3, "min count": 2, "use aligner": false, "wild type": {"coding": true, "reference offset": 534, "sequence": $CBS_TILE6_SEQ}}}
  The “Basic” mode was used (variant calling on R1 only), and variants to the unknown base N were filtered out.
4. satmut_utils v1.0.1-dev001: “-m 2 -q 30 -e 10 -s NNK”

### Generation of error correction validation datasets

The same accessory script used for generation of the benchmarking dataset was used for generation of four “sim” datasets. To estimate error correction parameters, we ran satmut_utils “call” on each NC library with the parameters “-m 1 -q 30 -e 10 -s NNK” to count false positive mismatches in the control alignments. satmut_utils “call” was also ran on an input source-matched, mutagenized library with the same parameters, except with a min count of 2 (-m 2). NC and mutagenized satmut_utils summary.txt files, along with the trimmed NC alignments, were used as inputs to the script. To complete each simulated dataset, satmut_utils “call” was ran on the output FASTQs, with the same parameters as NC libraries. Each simulated dataset (*N* = 4) comprised thousands of true positives (min 4850, max 7859) and thousands of false positives (min 4682, max 6463).

### Data postprocessing and error modeling

Custom R functions in summarization_utils.r and modeling_utils.r [76] were used to postprocess and model the resulting simulated datasets. The packages data.table [77], ggplot2 [78], cowplot [79], viridis [80], and ggsci [81] were used for data processing and graphics. The following packages were used for modeling: leaps [82], caret [83], e1071 [84], class [85, 86], randomForest [87], gbm [88], and glmnet [89].

Variant calls in each simulated dataset within the mutagenized target region, and with frequency < 0.3, were selected for modeling. Five classifiers were trained: gradient boosted machine (decision trees); generalized linear model (binomial family) with elasticnet regularization; *k*-nearest neighbors; random forest; and support vector classifier. Performance was evaluated by nested tenfold cross-validation (CV), selecting 20% of each fold’s training data for hyperparameter tuning with caret::train. Additionally, for *k*-nearest neighbors, the number of features was tuned in each fold with best subset selection (leaps::regsubsets) by fivefold CV, using between 3 and 10 features. For predictions of all models, a probability cutoff of 0.5 was used. The feature importance metric (mean decrease in accuracy) was determined by passing importance = TRUE during random forest training and subsequently calling randomForest::varImpPlot(type = 1, scale = TRUE).

### End-to-end analysis of growth-based MAVE data

For both *SUMO1* [4] and *HA* [30] datasets, FASTQs were downloaded from SRA using “fastq-dump –split-3 –origfmt.” The –origfmt flag is important so read names are single integers, compatible with the satmut_utils primer masking workflow.

The same preprocessed FASTQs input to satmut_utils were analyzed by the full DiMSum pipeline (WRAP, STEAM), version 1.2.11. For comparison, only STEAM (–startStage 4) was used for the post-processed, merged count files from satmut_utils. Both analyses used the following parameters:

`-u codon –mixedSubstitutions T –fitnessMinInputCountAll 10 –sequenceType coding`

To enable statistical inference with DiMSum [16], satmut_utils vcf.summary.txt files were post-processed using run_satmut_utils_to_dimsum.py [76], and per-sample count files were merged with the merge_dimsum_tables function in summarization_utils.r [76] for normalization to the wild-type count. All reference files for *SUMO1* and *HA* are available in the Supplementary repository [90] in the reference_files directory.

#### Analysis of SUMO1 MAVE data

No preprocessing on the FASTQs downloaded from SRA was performed before satmut_utils “call” with the following parameters:

“-r SUMO1.fa call -g SUMO1.gtf -k SUMO1.fa -m 1 -v -s NNK”

We ran the full DiMSum pipeline with the above parameters, and configured trimming of the first base of the amplicon to restore frame:

SUMO1_TILE2_SEQ = ”tGGACAGGATAGCAGTGAGATTCACTTCAAAGTGAAAATGACAACACATCTCAAGAAACTCAAAGAATCATACTGTCAAAGACAGGGTGTTCCAATGAATTCACTCAGGTTTCTCTTTGAGGGTCAGAGAATTGCTGATAATCATACTCCA”.

“–wildtypeSequence $SUMO1_TILE2_SEQ”.

#### Analysis of influenza WSN HA MAVE data

To preprocess dms_tools2 dual-barcoded reads for analysis with satmut_utils, a postprocessing script was used to convert reads to satmut_utils compatible format (run_dmstools2_to_satmut_utils.py) [76].

This script moves the UMI from the 5′ end of R2 to the 5′ end of R1 to concatenate both UMIs at the start of R1, enabling consensus deduplication with satmut_utils. Then, satmut_utils “call” was ran with the following parameters:

“-r WSN_HA.fa -p WSN_HA_primers.bed call -g WSN_HA.gtf -k WSN_HA.fa -m 1 -v -d -u "(?P<umi_1>[ATCGN]{16})"”

The full DiMSum pipeline was executed for each of the six HA amplicons and the above DiMSum parameters, but with inclusion of “–cutadaptCut5First 16” to trim the concatenated UMI from R1. DiMSum –wildtypeSequence arguments for each amplicon are provided in the Supplementary repository [90].

### Differential abundance analysis

Variant calls were filtered by several sequential steps prior to differential abundance analysis. First, variant frequencies were adjusted by subtracting the log_10_ variant frequency in the NC library from the frequency of corresponding variants in the mutagenized libraries. Then, candidate variants were selected in sequential order by the following criteria: (1) variant is within the mutagenized target region; (2) variant matches the NNK codon mutagenesis signature; (3) variant is a single-codon change; (4) SNP variant count ≥ 2 and MNP variant count ≥ 1; (5) no strong strand bias (RACE-like method only, nucleic acid strand count difference ≤ 64); (6) no variants with false positive RF predictions in all replicates (probability cutoff 0.49); (7) variant is observed in all replicates. Additionally, for amplicon data, one sequencing library with possible bottlenecking (gDNA replicate 3, Additional file 2: Fig. S4A) was dropped and replaced with the plasmid library sample to achieve three replicates. Amplicon method gDNA replicate 3 was warranted for exclusion as it formed its own cluster from other gDNA and cDNA replicates by hierarchical clustering analysis.

For filter 6, model training datasets were generated as described, and a RF model was trained on the following features: log_10_ frequency, variant type (SNP, di-nt MNP, tri-nt MNP), matches mutagenesis signature, substitution (e.g., A > G,T > C; six factor levels), upstream reference nt, downstream reference nt, R1 and R2 median supporting base qualities, R2 median supporting read position, R2 median supporting edit distance. For the RACE-like model, R1 and R2-specific features (base quality, read position, read edit distance) were additionally provided for each sample strand (R1 + , R1 − , R2 + , R2 −), along with the sample strand count difference. For read position and edit distance features, only R2 was used due to collinearity with the corresponding R1 features. RACE-like model training also required na.action = “na.roughfix” to handle NAs in training data due to count observations on only one sample strand.

To determine variants with differential abundance between cDNA (mRNA) and gDNA, we first normalized variant counts to the wild-type count at each position to produce a normalized frequency. We then used limma-trend [91] with empirical Bayes moderation and Benjamini–Hochberg multiple test correction to compare log frequencies between cDNA and gDNA. gDNA readout serves to normalize for library abundance and recombination efficiency of each variant. Relative changes thus reflect variant effects on population-wide, steady-state mRNA abundance.

### Replicate analysis

For Fig. 5E–G, variant calls were processed as described for differential abundance analysis with the exception of the last criterion (#6, variant observed in all replicates). Instead, variants observed in only one replicate were filtered out. For Fig. 5G, due to lower depth of coverage in RACE-like sequencing libraries, the median variant frequency of cDNA replicates was plotted for variants with a log_10_ frequency >  − 5.2 in amplicon gDNA libraries. This is the approximate limit of detection for the RACE-like method given the attained coverage.

### Comparison of library preparation methods

The theoretical number of possible calls in *CBS* tiles 2 and 4 (2676) was calculated empirically by counting all single SNPs, di-nt MNPs, and tri-nt MNPs that match a NNK mutagenesis signature for each codon in the *CBS* coding sequence. Unless otherwise noted, variant counts, frequencies, and cDNA-gDNA frequency difference (effect estimates) follow filtering as described in “Differential abundance analysis” and “Replicate analysis” and use the median for replicate summarization.

For mRNA abundance effect comparison (Additional file 2: Fig. S6B), variants determined significant in the amplicon method at FDR < 0.1 were assessed in RACE-like data. Only variants that were observed in all gDNA and cDNA replicates by both methods were compared.

### Analysis of tRNA abundance

Variants identified in the amplicon method at a FDR < 0.15 were used for analysis of tRNA abundance data to achieve better power for analysis. For mim-tRNAseq [55], the mean of counts was taken of the HEK293T duplicates. For Hydro-tRNAseq [56], HEK293 counts were used directly. For both datasets, anticodon abundance for codons with Crick wobble base pairing (A-G and C-T) were added to the dataset. Then, the sum of isodecoder counts was taken for each codon and log transformed. The difference between the log sum of counts (log ratio) was calculated between the alternate (variant) and reference codons and compared to the log fold changes determined by limma. Splines were fitted with R stats::lm and splines::bs with the parameters “knots = 0, degree = 2,” and the models were used to predict the response.

### Analysis of codon stability coefficient (CSC) data

The same set of variants used in analysis of tRNA abundance data was used to test for differences in CSC for the ORFome in HEK293T cells [52]. For Fig. 6B, the difference between the alternate codon (Alt.) and reference codon (Ref.) CSC was computed. For Fig. 6C, all pairwise distances of this difference between significant and non-significant variants (691 total variants, cDNA to gDNA log ratio) was determined to define the null distribution. Variants with a distance in the upper quartile of this null distribution are shown.

### Cell culture

HEK293T LLP iCasp9 Blast cells [31] were confirmed to be free of Mycoplasma and were cultured in Dulbecco’s modified Eagle’s medium (Thermo Fisher Scientific, 11995065) with 10% fetal bovine serum (Gibco) and 1% penicillin–streptomycin (Gibco, 15140122). Prior to recombination at passage 16, cells were selected for 1 week with 2 µg/mL doxycycline (Sigma-Aldrich, D3072) and 10 µM blasticidin (Gibco, A1113903) to enrich for cells with the integrated landing pad.

### CBS library cloning

A prior *CBS* variant library was used [10]. The *CBS* entry library was transferred into pDEST_HC_rec_bxb_v2, a vector containing Bxb1 recombination sites for the HEK293T LLP iCasp9 Blast landing pad line, by a Gateway LR II reaction (Thermo Fisher Scientific, 11–791-020) following the manufacturer’s recommendations. 1.5 µL of LR reaction was transformed into 25-µL Endura Electrocompetent cells (Lucigen, 60242), plated on Nunc Square Bioassay dishes, scraped in 6 mL LB Miller broth, and 3 mL resuspension was processed with the ZymoPURE II Plasmid Maxiprep Kit (Zymo Research, D4203). Library size was estimated at ~ 540,000 species, or ~ 30-fold coverage of each possible SNP or MNP variant in the *CBS* coding sequence.

### Stable expression of CBS variant library

Twenty micrograms of the *CBS* variant library (in pDEST_HC_Rec_Bxb_V2), along with an equal mass of Bxb1 recombinase (pCAG-NLS-HA-Bxb1) was transfected into three 15-cm dishes of HEK293T LLP iCasp9 Blast cells (passage 18, 65% confluency) using Lipofectamine 3000 (Thermo Fisher Scientific, L3000008), with volumes scaled based on 3.75 µL reagent per 6 wells. Twenty-four hours later, cells were split 1:2 into 15-cm dishes. Forty-eight hours after transfection, at near full confluency, 2 µg/mL doxycycline and 10 nM AP1903 (MedChemExpress, HY-16046), both solubilized in DMSO, were added for negative selection of non-recombined cells. The next day, dead cells were removed and recombined cells were grown out for an additional 2 days with fresh media containing doxycycline and AP1903. Cells were recovered for 1 day by growth in media without doxycycline and AP1903. Transcription was induced with 2 µg/mL doxycycline for 24 h; cells were stimulated with fresh media for 3 h, and then harvested at 95% confluency.

### gDNA extraction

gDNA was extracted from approximately 3 to 4 million cells with the Cell and Tissue DNA Isolation Kit (Norgen Biotek Corp, 24700), including RNaseA treatment and eluting in 200 µL warm elution buffer.

### RNA extraction and cDNA synthesis

Approximately 3–4 million cells were solubilized with 1 mL QIAzol (Qiagen, 79306) and 0.2 mL chloroform in 5PRIME Phase-Lock Gel heavy tubes (QuantaBio, 2302830), according to the manufacturer’s recommendations. RNA was precipitated at − 20 °C following the addition of 2 µL GlycoBlue (Thermo Fisher, AM9515) and 2.5 volumes of cold absolute ethanol. RNA was washed once with cold 70% ethanol then resuspended in 30 µL water. Ten micrograms total RNA was treated with DNaseI (NEB, M0303), then re-purified by the RNA Clean and Concentrator Kit (Zymo Research, R1015) and eluted in 15 µL water. RNA quality was assessed with the Bioanalyzer Eukaryotic RNA Pico kit (Agilent, 5067–1513). For each of six reactions, 2.5 µg DNaseI-treated total RNA was denatured at 65 °C for 5 min followed by RT primer annealing at 4 °C for 2 min, using 2 pmol pDEST_HC_Rec_Bxb_v2_R primer specific for the landing pad. See Additional file 3: Table S3.

Primed total RNA was included in six 20 µL SuperScript IV cDNA synthesis reactions (Thermo Fisher, 18090010) with SUPERase-In RNase inhibitor (Thermo Fisher, AM2696), and first-strand cDNA was synthesized by incubating at 55 °C for 1 h, followed by RT inactivation at 80 °C for 10 min. RNA was digested with addition of 5 U RNaseH (NEB, M0297) to the first-strand cDNA synthesis reaction and incubation at 37 °C for 20 min. One reaction was saved for amplicon library preparation, while the other five were saved for RACE-like (Anchored Multiplex PCR) library preparation.

### Amplicon library preparation

See Additional file 3: Table S3 for primers used in PCR1 and PCR2 of amplicon method library preparation, outlined below.

#### Landing pad amplification (PCR1)

2.5 µg of gDNA was amplified with Q5 polymerase (NEB, M0491) for 14 cycles in each of six 50 µL PCR reactions with 500 nM landing-pad-specific primers (pDEST_HC_Rec_Bxb_v2_F, pDEST_HC_Rec_Bxb_v2_R) flanking the entire *CBS* insert (~ 1.7 kb), and including the high GC enhancer reagent. The cycling parameters were as follows: initial denaturation at 98 °C for 30 s; 3-step cycling with denaturation at 98 °C for 10 s, anneal at 65 °C for 30 s, extension at 72 °C for 1 min; final extension at 72 °C for 2 min.

Products were pooled, resolved on a 0.8% agarose/TAE gel, visualized with 1 × SYBR Gold, and extracted from the gel using the Macherey–Nagel Nucleospin Gel and PCR Cleanup Kit (Takara, 740609) with 15–25 µL 70 °C elution buffer.

#### Coding sequence amplification (PCR2)

Five hundred picograms of the gDNA and cDNA PCR1 products (landing pad insert) was amplified for each of two CBS tiles (CBS_2_v2, CBS_4_v2 primer pairs) in a 50 µL NEB Q5 reaction (NEB, M0491) with high GC enhancer for 8 cycles, following the same cycling parameters as for PCR1.

#### Illumina adapter addition (PCR3)

Products for tile 2 and 4 amplicons were cleaned up with the Nucleospin Gel and PCR Cleanup Kit (Takara, 740,609), eluted in 30 µL 70 °C buffer, and then mixed together at equal volumes (5 µL each) and input into a final NEB Q5 reaction for 8 cycles with the same formulation as PCR2 but using NEBNext Multiplex Oligos for Illumina Dual Index Primers Set 1 (NEB, E7600S) according to the manufacturer’s recommendations (65 °C annealing). Final library was purified with the Nucleospin Gel and PCR Cleanup kit and eluted in 30 µL 70 °C buffer.

### RACE-like library preparation

Anchored Multiplex PCR libraries were generated with modifications following the initial strategy [21]. Briefly, for cDNA libraries, double-strand cDNA was first synthesized, and gDNA and double-strand cDNA inputs were sheared prior to library preparation. Libraries were prepared using the ArcherDX, Inc. (now Invitae) LiquidPlex library preparation kit with a custom *CBS* primer assay. See Additional file 3: Table S4 for primer sequences.

#### Second-strand cDNA synthesis

Second-strand cDNA synthesis was carried out with 1st-strand cDNA from each of five reactions converting 2.5 µg total RNA and digesting with 5 U RNaseH. 2 pmol landing-pad-specific forward primer (pDEST_HC_Rec_Bxb_v2_F) and 1 µL Q5 polymerase (NEB, M0491) were added to 20 µL of each 1st-strand cDNA reaction and incubated as follows: 95 °C denaturation for 30 s, followed by three cycles of (1) 55 °C anneal for 30 s and, (2) 72 °C extension for 3 min, for a total of three linear primer extension cycles. Reactions were pooled and cleaned up with the Nucleospin Gel and PCR Cleanup kit with 1:1 buffer NTI dilution and elution with 15 µL 70 °C elution buffer.

#### DNA fragmentation

gDNA was extracted from 5 million cells with the Cell and Tissue DNA Isolation Kit (Norgen Biotek Corp, 24700), including RNaseA treatment and eluting in 200 µL warm elution buffer. gDNA and double-strand cDNA were fragmented using the Covaris S2 with the following parameters: microTUBE AFA Fiber Snap-Cap, 10% duty cycle, 5 intensity, 200 cycles per burst, 1 min treatment. Sheared DNA inputs were brought up to 50 µL with ultrapure water.

#### PCR enrichment and library preparation

Libraries were prepared from 1–1.5 µg sheared gDNA or double-stranded cDNA according to the manufacturer’s recommendations (ArcherDX, PRO027.4), using 15 cycles for both PCR1 and PCR2 with custom *CBS* primers (Additional file 3: Table S4).

### Quantification and size selection of final libraries

Final libraries were quantified using the KAPA Library Quantification Kit for Illumina (Roche, KK4873) according to the manufacturer’s recommendations, using 1:10,000 dilution of libraries and size correction with an average fragment size of 300 nt. qPCR was performed on the Applied Biosystems ViiA 7 instrument. The final pool was size-selected using Sage Bioscience BluePippin 2% gel (marker V1 and/or V2) to select fragments from 200 to 400 bp. Following size selection, the final pool was quantified by both the High Sensitivity DNA Kit (Agilent, 5067–4626) and Qubit High Sensitivity DNA assay (Thermo Fisher, Q32851).

### Preparation of negative control libraries

Negative control libraries and mutagenized libraries used for estimating true variant frequencies were prepared from various sources (HEK293T total RNA, plasmid DNA) with several methods. See protocols for each library under the GEO submission. Primer sequences for all *CBS* amplicons are provided in Additional file 3: Table S5.

### Next-generation sequencing

Libraries were sequenced 2 × 150 (paired-end) at MedGenome, Inc. on the Illumina HiSeq X platform. RACE-like (Anchored Multiplex PCR) libraries were re-sequenced on the Illumina NovaSeq 6000, and FASTQs were concatenated prior to analysis. Five to ten percent PhiX was included during sequencing.

## Supplementary Information

**Additional file 1.** Comments on satmut_utils algorithmic design, benchmarking, and error correction [95–105].

**Additional file 2: Fig. S1.** satmut_utils design. A) Comparison of amplicon and RACE-like library preparation methods. **Fig. S2.** Comparison of variant callers for nucleotide changes. A) satmut_utils count accuracy. **Fig. S3.** Library preparation quality control. **Fig. S4.***CBS* variant frequency correlations for amplicon and RACE-like methods. **Fig. S5.***CBS* variant effects by the amplicon method. **Fig. S6.***CBS* variant effects by the RACE-like method. **Fig. S7.** Terminal amplicon bias in variant effect for the RACE-like method.

**Additional file 3: Table S1.** satmut_utils 'call' error correction features. **Table S2.** Variants with differential RNA abundance by amplicon and RACE-like methods. **Table S3.** pDEST_HC_Rec_Bxb_v2_CBS PCR1 and PCR2 primers for amplicon method. **Table S4.** CBS tail-less primers for RACE-like, Anchored Multiplex PCR method. **Table S5.** CBS primers for negative control library amplification.

**Additional file 4.** Review history.

## Data Availability

The satmut_utils Python package is released under a GPLv3 license and is compatible with Linux and Mac OS X operating systems. Source code, installation instructions, and documentation are available on GitHub [92] and Zenodo [76]. All analyses used a development version of satmut_utils (v1.0.1-dev001) containing accessory scripts for secondary analysis [76]. Supplementary information [90] and curated reference files [93] are available online. Raw sequencing data is available in GEO under accession GSE201057 [94], and simulated datasets are available on Zenodo [25].
