# Supplementary material for: satmut_utils: a simulation and variant calling package for multiplexed assays of variant effect
Source: Genome Biol. 2023 Apr 20;24:82. doi: 10.1186/s13059-023-02922-z (PMC10116734; doi:10.1186/s13059-023-02922-z)
Supplement: Supplementary file 2 — Additional file 2: Fig. S1. satmut_utils design. A) Comparison of amplicon and RACE-like library preparation methods. Fig. S2. Comparison of variant callers for nucleotide changes. A) satmut_utils count accuracy. Fig. S3. Library preparation quality control. Fig. S4.CBS variant frequency correlations for amplicon and RACE-like methods. Fig. S5.CBS variant effects by the amplicon method. Fig. S6.CBS variant effects by the RACE-like method. Fig. S7. Terminal amplicon bias in variant effect for the RACE-like method. [file 13059_2023_2922_MOESM2_ESM.docx]

**Additional file 2**


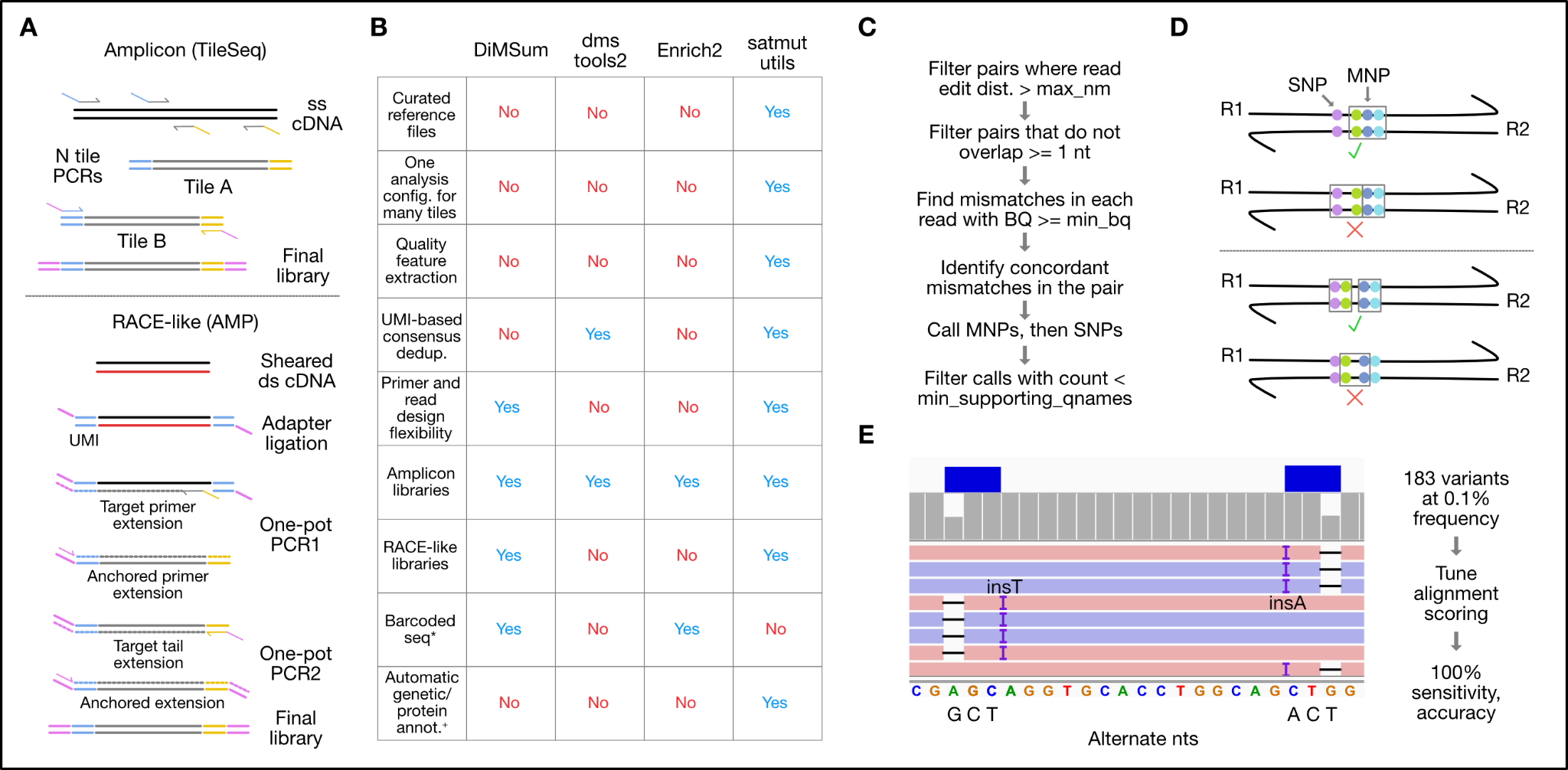


**Fig. S1: satmut_utils design. A) Comparison of amplicon and RACE-like library preparation methods.** Pink segments indicate the first half of the adapters (Illumina P5/P7 sites). Blue and yellow segments indicate the rest of the adapter, containing read primer sites and possibly a unique molecular index (UMI). TileSeq is an amplicon method [[4]](https://paperpile.com/c/nlEHL5/Gjnsk). Anchored Multiplex PCR (AMP) [[21]](https://paperpile.com/c/nlEHL5/wtkbY) is a RACE-like method. **B) Feature comparison of MAVE variant callers.** Blue text indicates software utility/flexibility while red text indicates unsupported features. *Barcoded-seq refers to estimating variant abundance through counting a linked unique barcode. ^+^Codon and protein change annotations are reported with correct coding sequence positions, requiring no manual configuration of offsets. **C) Variant calling algorithm.** Read pairs are filtered with max edit distance (dist., nm) and min base quality (BQ) parameters before finding concordant mismatches (same base call in R1 and R2). **D) Schematic of MNP calling algorithm.** Gray boxes denote the MNP span. In cases where there are multiple mismatches within the window, satmut_utils prioritizes contiguous mismatch runs. In one hypothetical case, a mismatch precedes a contiguous run of three mismatches. The compact run is called as a tri-nt MNP, and the preceding mismatch is called as a SNP. In a second case, two compact runs- each with two mismatches- are spaced by one base matching the reference. Each run is called as a di-nt MNP. **E) Example of tri-nt MNPs aligning as InDels.** Under default bowtie2 alignment parameters (--rdg/--rfg 5,3), MNPs may be aligned as InDels. After adjustment of the scoring parameters (--rdg/--rfg 6,4), MNPs aligned as contiguous mismatches (Methods).

**
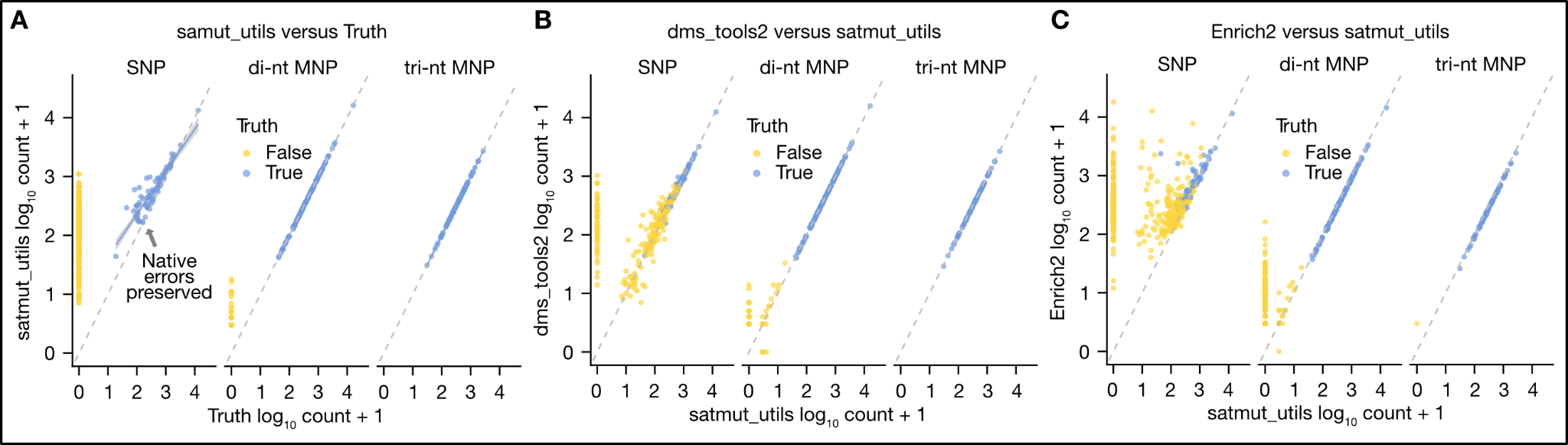
**

**Fig. S2: Comparison of variant callers for nucleotide changes. A) satmut_utils count accuracy.** Simulated truth counts are compared to satmut_utils, dms_tools2, and Enrich2 reported counts. Deviation for SNPs is due to preservation of native errors during satmut_utils simulation. Dotted gray lines indicate equivalence. Blue lines show the slope of a linear regression fit between truth and observed counts, with 95% confidence intervals in gray. **B) satmut_utils comparison to dms_tools2.** Counts for true (blue) and false positive (yellow) variants are shown. **C) satmut_utils comparison to Enrich2.** Counts are shown as in B. Higher false positive variants for Enrich2 is partly due to use of its Basic mode, which uses only R1 for variant calling. Enrich2 Overlap mode led to a high proportion of unresolved calls, which precluded analysis (see Additional file 1).

**
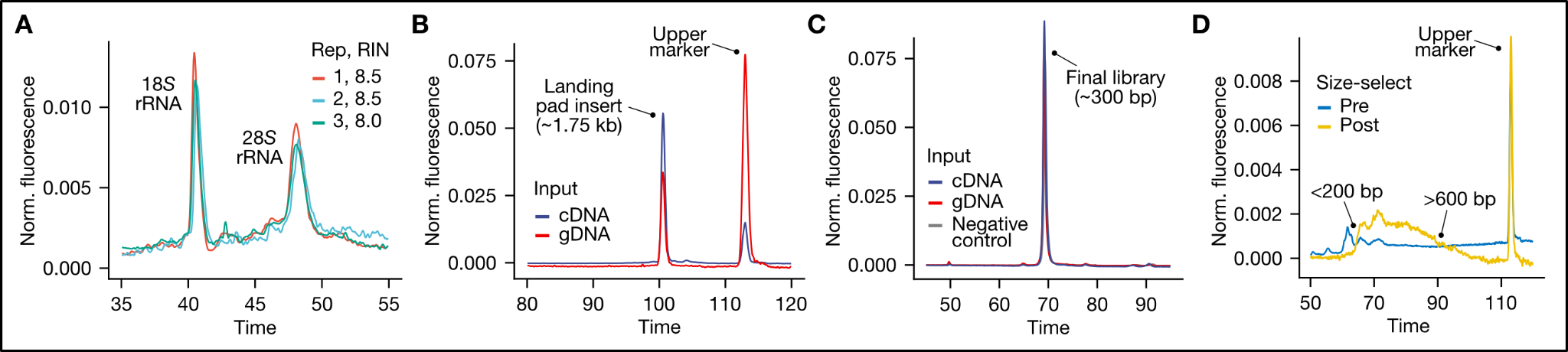
**

**Fig. S3: Library preparation quality control.** For all panels, normalized (Norm.) fluorescence was computed by dividing by the sum of fluorescence across the displayed Time range. Panel A shows results from the Agilent Eukaryotic RNA Pico kit. Panels B-D show results from the Agilent High Sensitivity DNA kit. For panels B and C, traces indicate the mean of biological replicates. **A) Quality of biological replicate total RNA**. DNaseI-treated total RNA was assayed and the 18*S* and 28*S* rRNA peaks for each replicate (Rep), with RNA integrity number (RIN), are shown. **B) Confirmation of intermediate products for the amplicon method**. PCR1 was performed to enrich the landing pad insert (*CBS* coding sequence) from gDNA and cDNA prior to PCR2 for tiled amplicons. **C) Final library confirmation for the amplicon method**. Analysis of final libraries (PCR3) confirmed a specific product of the expected size (~150 bp insert plus adapters). **D) Final library confirmation and size-selection for the RACE-like method**. Final gDNA and cDNA libraries were pooled and assayed before and after size-selection (Methods). Exclusion of incompletely-adapted library and short (<50 bp) or long (>450 bp) inserts is denoted.

**
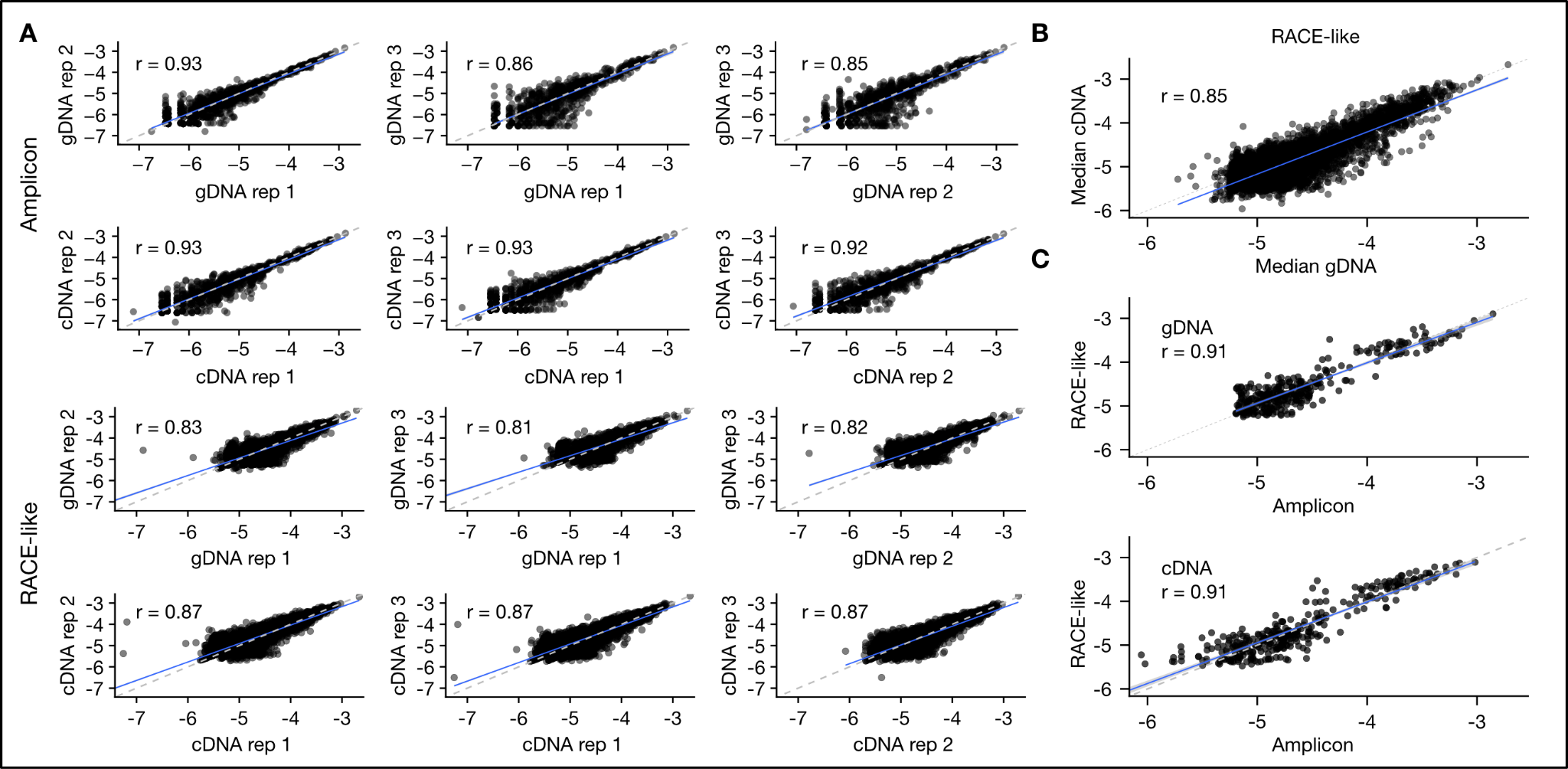
Fig. S4**: ***CBS* variant frequency correlations for amplicon and RACE-like methods**. In all panels, log_10_ variant frequencies are plotted after filtering out variants found in only one gDNA or cDNA library replicate. Pearson's correlation coefficient (r) is indicated. Grey dotted lines indicate equivalence. Blue lines are a linear regression fit, with gray shading indicating 95% confidence intervals. **A) Biological replicate reproducibility**. gDNA and cDNA variant frequencies are shown for replicate cell lines independently recombined with the *CBS* variant library. **B) Correlation between gDNA and cDNA for RACE-like libraries**. The median frequency was computed among gDNA and cDNA replicates prior to comparison. **C) Variant frequency correlation between methods**. Due to lower depth of coverage for RACE-like libraries, variants with log_10_ gDNA frequencies greater than -5.2 were selected for comparison to the RACE-like method (Anchored Multiplex PCR) [[21]](https://paperpile.com/c/nlEHL5/wtkbY). Replicate summarization used the median. Top panel compares gDNA libraries, and bottom panel compares cDNA libraries.

**
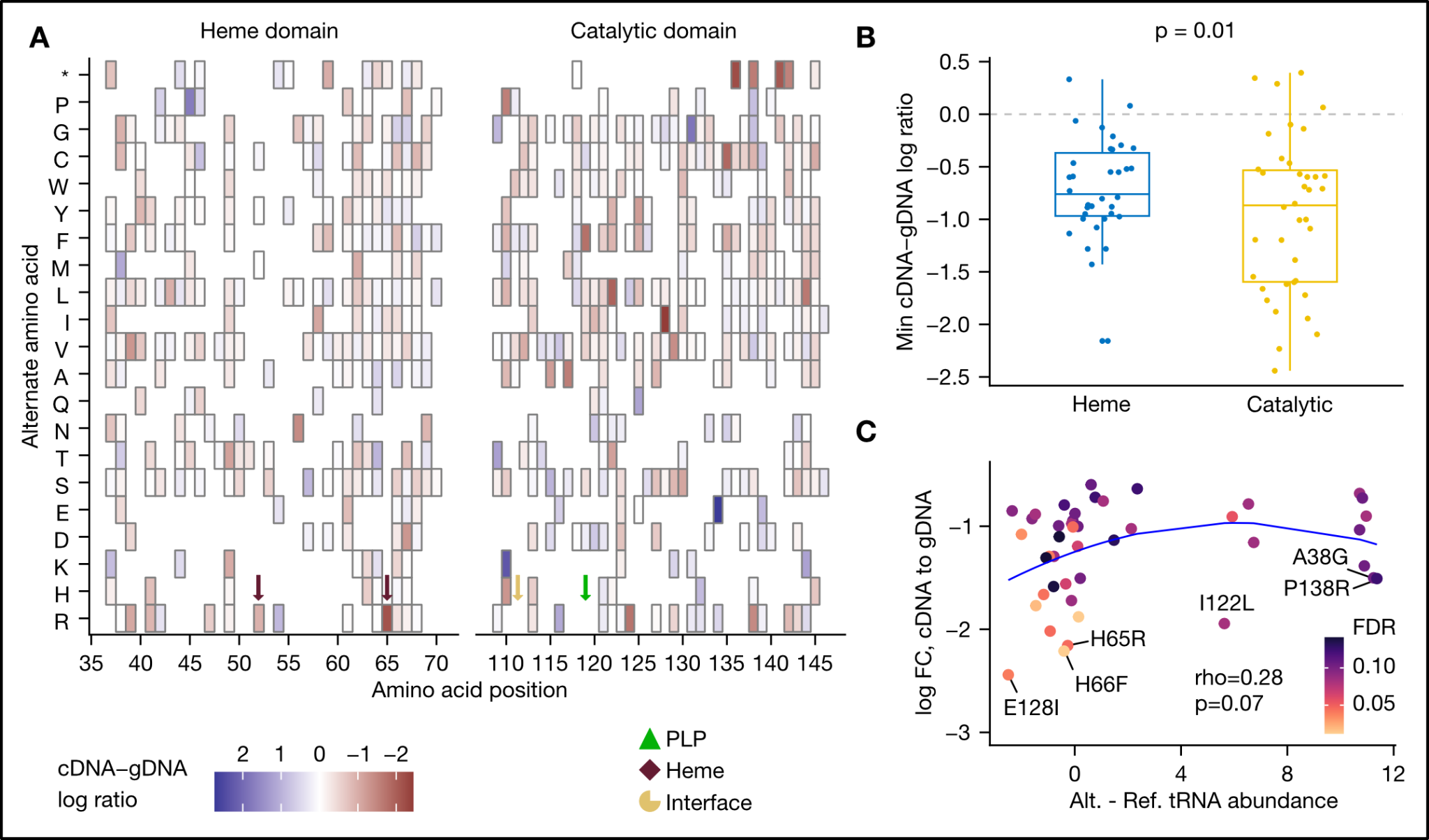
**

**Fig. S5: *CBS* variant effects by the amplicon method**. **A) CBS variant effect map**. Normalized log frequencies for variants detected in more than one replicate and leading to the same amino acid change were summarized; fill is the mean log ratio between cDNA and gDNA. Arrows demarcate residues with heme binding (C52, H65), pyridoxal-5’-phosphate (PLP) binding (K119), or a location at the dimerization interface (111-112). **B) Higher variance of effects in the catalytic domain**. The minimum log ratio between cDNA and gDNA was computed in each domain and tested by a rank-based Brown-Forsythe Levene-type test. **C) tRNA abundance correlation with magnitude of variant effect**. The log fold change (logFC) for significant variants (FDR < 0.15) is compared to the log ratio of tRNA abundance (Alt. codon - Ref. codon) measured for HEK293T by mim-tRNAseq [[55]](https://paperpile.com/c/nlEHL5/4XhGc). Blue line is a polynomial spline with a knot at 0. Confidence intervals were omitted for clarity. Spearman rank correlation (rho) and p-value are shown.

**
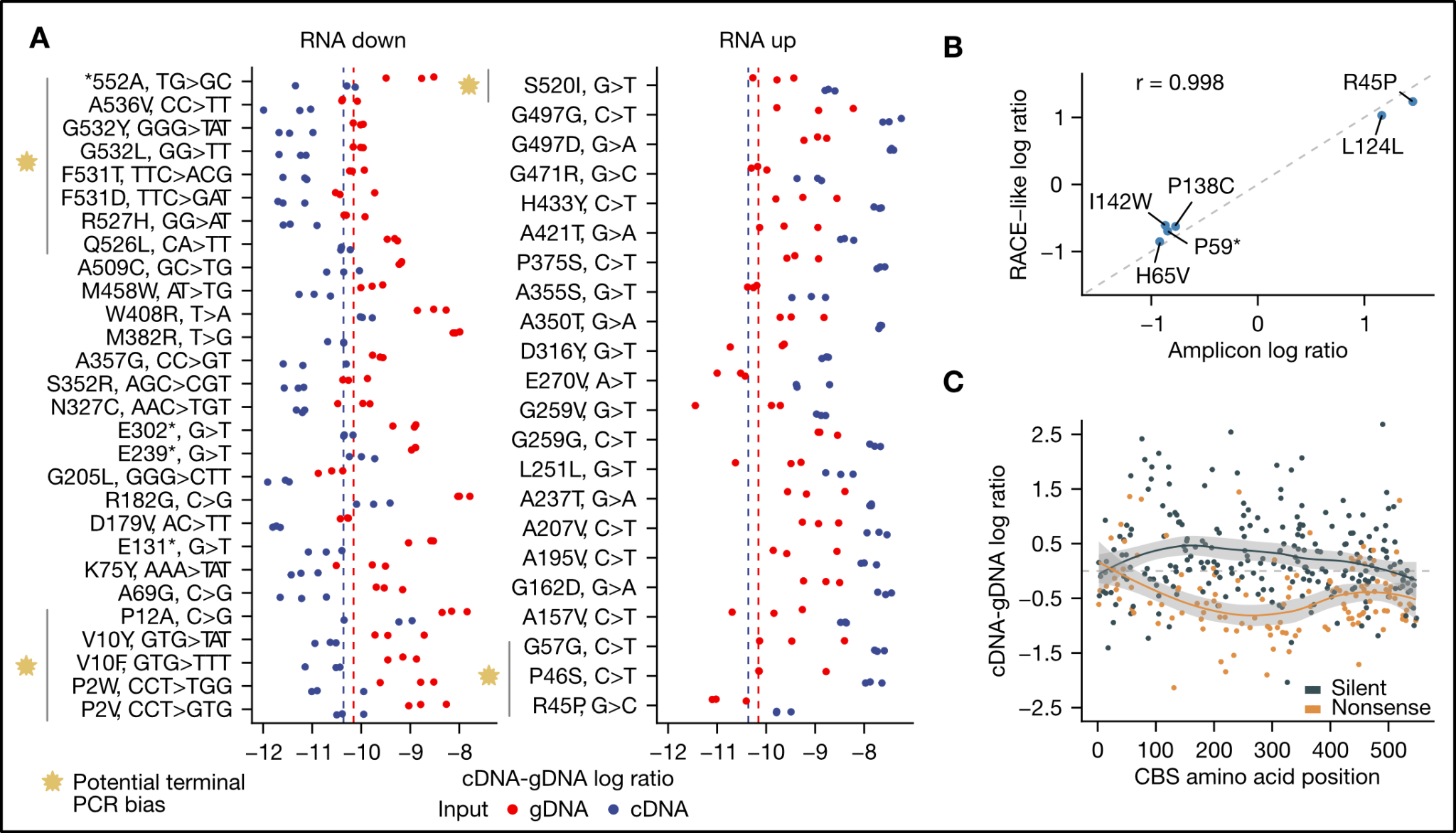
**

**Fig. S6**: ***CBS* variant effects by the RACE-like method.** In all panels, a gray dotted line indicates no change in variant effect or comparative metrics. **A) Differential variant abundance.** y-axis labels denote the amino acid change and the nucleotide substitutions for significant variants (FDR < 0.1). Red and blue dotted lines represent the median of each input source for all variants. Variants with a potential bias are flagged (Additional file 2: S7, Additional file 1). **B) Correlation of variant effects between amplicon and RACE-like methods.** Variants significant by the amplicon method (FDR < 0.1) are plotted if detected in all replicates by the RACE-like method. Pearson’s correlation coefficient is shown for the log ratio between cDNA and gDNA. **C) The RACE-like method reveals nonsense variant effects depend on location**. The median log frequency of variants detected in more than one replicate was calculated, then the log ratio between cDNA and gDNA for silent and nonsense variants was plotted across the *CBS* coding region. Lines represent local regression fit, with 95% confidence intervals in shaded gray.


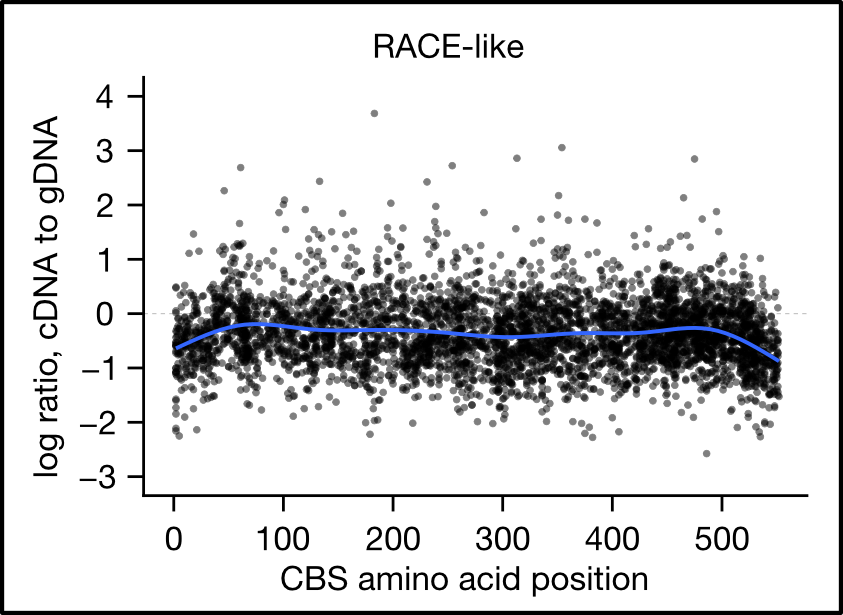


**Fig. S7: Terminal amplicon bias in variant effect for the RACE-like method.** Variants in the terminal tiles adjacent to vector sequence may have lower variant effect estimates. This could be due differential processing of gDNA and cDNA required for library preparation. Blue line represents a local regression fit. See Additional file 1 for an explanation.
