# Supplementary material for: satmut_utils: a simulation and variant calling package for multiplexed assays of variant effect
Source: Genome Biol. 2023 Apr 20;24:82. doi: 10.1186/s13059-023-02922-z (PMC10116734; doi:10.1186/s13059-023-02922-z)
Supplement: Supplementary file 4 — Additional file 4. Review history. [file 13059_2023_2922_MOESM4_ESM.docx]

Review History

**First round of review**

**Reviewer 1**

**Were you able to assess all statistics in the manuscript, including the appropriateness of statistical tests used?** Yes, and I have assessed the statistics in my report.

**Comments to author:**

The paper submitted by Hoskin and colleagues presents satmut_utils a new tool for variant calling and analysis on MAVE experiments.

The method has been benchmarked on a mutagenesis data on CBS obtained with two preparation methods.

Overall the work is well written and easy to follow. In my opinion the paper could be considered for publication after addressing the

minor issues reported below.

Minor Revisions

1) The definition of the measure of performance used for benchmarking the method should be described either in the methods section or in supplementary materials.

- Can you formally define the "count accuracy" that is discussed in the caption of Fig 1?

- Is the "count accuracy" different from the accuracy reported in Fig 2?

- Are the Precision and Recall calculated on the simulated date using their standard definition?

2) The benchmarking of the method has been performed on a unique gene (CBS).

- Can you comment about the generalization of you results?

- Do you expect that satmut_utils should be optimized when applied to the data on different gene?

3) According to your results the implemented RF method is robust and shows similar performance

when using different protocols for generating negative controls. In Fig. 2C you reported the

performance of the method obtained on data from different protocols.

- Is this meaning that in the cross-validation procedure you trained on platform A and tested on platform B?

- Can you briefly describe what are the top 15 important features used for the classification?

**Reviewer 2**

**Were you able to assess all statistics in the manuscript, including the appropriateness of statistical tests used?** Yes, and I have assessed the statistics in my report.

**Comments to author:**

The manuscript describes a specialized variant caller and set of simulation utilities (satmut_utils) for use in MAVE experiments. The authors use these simulation utilities to conduct a benchmarking comparison between their variant caller and other variant callers for MAVEs. They also apply their variant caller to two MAVE libraries for mutations in cystathionine beta-synthase (CBS) in order to determine the effects of these mutations on mRNA abundance.

The article is of appropriate impact and breadth of interest for Genome Biology due to the strong need for software to analyze MAVE experiments. The strategy for the variant caller, and particularly the treatment of multinucleotide polymorphisms and the use of the random forest modeling of sequencing errors, is sound, necessary to correctly account for differences between traditional sequencing efforts and typical MAVE library designs, and the authors show improved performance over existing methods.

However, the application to CBS, while biologically very interesting, is an unusual type of MAVE (assaying effects of coding changes on mRNA level rather than changes on protein function, growth rate, or protein stability as in a more typical MAVE) and the paper describing a general purpose computational tool should probably demonstrate the performance of that tool in a more typical use case, as well as provide comparisons with other tools on an end-to-end empirical dataset.

Moreover, the analysis of CBS is also marred by certain analytical choices as well as the disappointing lack of quantitative agreement between the measurements made by the two library designs in the region in which they overlap. The authors treat the analysis as one of detecting whether library variants are at different frequencies when measuring cDNA vs. genomic DNA. The difficulty introduced by this choice is that the frequency of any particular variant in the cDNA depends on the mRNA levels produced by the other variants in that library and thus does not describe a biological characteristic of the variant. For example, if most variants reduce mRNA levels, a variant that has no effect on mRNA levels will increase in frequency in the cDNA relative to the genomic DNA. The standard solution to deal with this problem is to normalize the change in frequency for each variant by the change in frequency of the wild-type sequence (i.e. calculate the enrichment ratio), which in the context of the CBS has the interpretation of estimating the mRNA level produced by a variant as a fraction of the wild-type mRNA level, a quantity that can be estimated consistently regardless of library composition. This analysis of enrichment (or sometimes log enrichment) is what is done in the competing DiMSum and Enrich2 packages and what should be done here.

A final comment concerns the applicability of the satmut_utils software to varying MAVE library designs. Specifically, the software is specialized for considering libraries similar to NNS designs but cannot accommodate barcodes or libraries with multiple mutations as would be produced by error prone PCR. This limitation should be made clearer to the reader in the abstract / introduction so that a potential reader will know quickly whether the software described here is able to accommodate their experimental data.

Minor comments:

Page 5 Line 33 "DimSum only annotates standard amino acid changes" The authors should clarify the claim here. DimSum works on nucleotide data as well (e.g. tRNAs), can handle NNS mutagenesis, and can handle multiple amino acid or nucleotide changes in a single read. Similarly pg 9 Line 17, why can't you run DimSum in nucleotide mode to get the nucleotide annotations?

Page 10 Line 57 "significantly" implies a statistical test. Evidence is strong here, but no test done. Suggest synonym.

Figure 1C Colored ticks on y axis not explained.

Figure 1D The SNP panel does not display the overall distributions of errors very well because all you can see is the high outliers. I suggest showing log (observed/true).

Figure 5B What is the p-value for the Spearman correlation?

Pg. 18 line 54. The integration with the previous DMS study should be improved. This study provides data capable of estimating defects in mRNA levels of variants relative to the wild-type. Is a substantial mRNA defect sufficient to produce a fitness defect in the yeast complementation assay? How does this impact the interpretation of the previous results on mRNA stability and tRNA abundance?

**Authors Response**

**Point-by-point responses to the reviewers’ comments:**

Reviewer #1: The paper submitted by Hoskin and colleagues presents satmut_utils a new tool for variant calling and analysis on MAVE experiments. The method has been benchmarked on a mutagenesis data on CBS obtained with two preparation methods. Overall the work is well written and easy to follow. In my opinion the paper could be considered for publication after addressing the minor issues reported below.

We thank reviewer #1 for their positive assessment of our manuscript.

1) The definition of the measure of performance used for benchmarking the method should be described either in the methods section or in supplementary materials.

- Can you formally define the "count accuracy" that is discussed in the caption of Fig 1?

In Figure 1D, the count accuracy quantified the percent deviation between the observed count and the expected (truth) count for simulated variants. Per reviewer #2 comments, we changed this metric to the log (observed/expected) to be more transparent in its calculation and better display results.

- Is the "count accuracy" different from the accuracy reported in Fig 2?

In Figures 2B-C, accuracy for a given variant is defined as the sum of true positives and true negatives divided by the sum of all calls- true positives (TP), false positives (FP), true negatives (TN), and false negatives (FN).

- Are the Precision and Recall calculated on the simulated date using their standard definition?

Precision and recall were calculated using their standard definition, i.e. Precision=TP/(TP+FP) and Recall=TP/(TP+FN).

2) The benchmarking of the method has been performed on a unique gene (CBS).

- Can you comment about the generalization of you results?

- Do you expect that satmut_utils should be optimized when applied to the data on different gene?

We thank the reviewer for these two questions. We think that it is essential for satmut_utils to generalize to other contexts and genes. In our unpublished ongoing work, we have generated a variant library in catechol-*O*-methyltransferase (*COMT*) generated by silicon-based DNA synthesis (Twist Biosciences). While this study will focus on variant effects on *COMT* expression, to address the question of generalization we first constructed another ‘sim’ dataset for *COMT*. We found comparably high performance of the random forest model, despite estimating parameters from negative and positive control libraries produced by an orthogonal mutagenesis method, library preparation experiment, and sequencing platform. Accuracy by 10-fold nested cross-validation yielded a mean accuracy of 0.999 +/- 0.002 (s.d). As with ‘sim’ datasets for *CBS*, this performance is likely optimistic (Additional File 1), but highlights the power of satmut_utils ‘sim’ and resulting error models to learn error signatures within each MAVE experiment. Importantly, the high performance achieved in this application to a new gene did not require any specific optimization beyond what is included in our Methods.

Second, we applied satmut_utils to analyze cDNA (mRNA) versus gDNA abundance for the variant library in *COMT*, using the same satmut_utils parameters as in the analysis of *CBS*. We found satisfactory correlation between gDNA and cDNA frequencies (**Figure R1A**). We also recapitulated our finding of an association between mRNA abundance effect and the difference in codon stability coefficient between alternate and reference codons (**Figure R1B,C**).

Third, we used satmut_utils to quantify variant frequencies in two additional MAVEs from the influenza *HA* gene and human *SUMO1* gene. These new results are described in the revised manuscript and are detailed below in our response to a specific comment from reviewer #2. Taken together, our analysis indicates satmut_utils performs well in processing data from different genes and sequencing methodologies, using equivalent variant calling parameters.

**Figure R1**: **Application of satmut_utils to a catechol-*O*-methyltransferase (*COMT*) variant library**. **A)** Correlation between gDNA and cDNA (mRNA) abundance for 2173 *COMT* variants. The median log_10_ frequency of technical replicates (N=3) was calculated and variants below a frequency cutoff of log_10_ -5.5 were filtered out. Pearson correlation coefficient (r) is shown. **B,C)** Association between *COMT* mRNA abundance effect and codon stability measurements. Variants with differential abundance at a FDR < 0.01 were compared to codon stability coefficient scores measured by either Wu et al. 2019 or Narula et al. 2019.

3) According to your results the implemented RF method is robust and shows similar performance when using different protocols for generating negative controls. In Fig. 2C you reported the performance of the method obtained on data from different protocols.

- Is this meaning that in the cross-validation procedure you trained on platform A and tested on platform B?

In Figure 2C, we used cross-validation to assess model performance within each ‘sim’ dataset (platform). To determine performance on independent ‘sim’ datasets derived from negative control libraries sequenced in different experiments, labs, organisms, and platforms, we carried out the analysis in Figure 2E. In this figure, we trained on a ‘sim’ dataset from one negative control library and tested on all other ‘sim’ datasets from other negative control libraries. That is, Figures 2C and 2D test performance within each ‘sim’ dataset, while the results in Figure 2E show performance across independent ‘sim’ datasets.

- Can you briefly describe what are the top 15 important features used for the classification?

We thank the reviewer for this suggestion. The top fifteen important features to the random forest model included features such as the frequency of the variant, the median base quality supporting the variant call, whether the variant is a MNP or SNP, and whether the variant matches the NNK mutagenesis signature. Other less-intuitive but important features were the identity of the substitution (e.g. A>G, T>C), the position of the variant in read 2, the edit distance of read 2, and the identity of the upstream and downstream nucleotide (where G in these positions was informative, possibly because GC-rich sequences may be error-prone). We included a list of all features and their importance metrics in the new Supplementary Table 1.

Reviewer #2: The manuscript describes a specialized variant caller and set of simulation utilities (satmut_utils) for use in MAVE experiments. The authors use these simulation utilities to conduct a benchmarking comparison between their variant caller and other variant callers for MAVEs. They also apply their variant caller to two MAVE libraries for mutations in cystathionine beta-synthase (CBS) in order to determine the effects of these mutations on mRNA abundance.

The article is of appropriate impact and breadth of interest for Genome Biology due to the strong need for software to analyze MAVE experiments. The strategy for the variant caller, and particularly the treatment of multinucleotide polymorphisms and the use of the random forest modeling of sequencing errors, is sound, necessary to correctly account for differences between traditional sequencing efforts and typical MAVE library designs, and the authors show improved performance over existing methods.

We thank reviewer #2 for their thorough review and positive assessment of our study.

However, the application to CBS, while biologically very interesting, is an unusual type of MAVE (assaying effects of coding changes on mRNA level rather than changes on protein function, growth rate, or protein stability as in a more typical MAVE) and the paper describing a general purpose computational tool should probably demonstrate the performance of that tool in a more typical use case, as well as provide comparisons with other tools on an end-to-end empirical dataset.

We thank the reviewer for this suggestion. In the revised manuscript, we have provided end-to-end analyses for two additional MAVEs that assayed coding variant effects on protein function (human *SUMO1* from Weile and Sun et al. 2017; and influenza *HA* from Doud and Bloom 2016). For both use cases, we compared the performance of satmut_utils with the most recently published MAVE analysis software (DiMSum). These new results are described in a new section in the manuscript titled “End-to-end analysis of growth-based MAVE data” (now Figure 4).

Briefly, we found that the choice of variant calling method had an appreciable impact on the total number of variant calls made as well as the derived fitness estimates. Importantly, satmut_utils showed greater correlation between biological replicates for both variant quantification and the resulting fitness estimates. Further, a greater separation in fitness estimates between silent, missense, and nonsense variants was achieved with satmut_utils compared to DiMSum in one of the assays.

Our analyses establish the flexibility of satmut_utils in analyzing growth-based MAVE data. Importantly, we have now developed and provided pre- and post-processing scripts for design conversion between satmut_utils, dms_tools, DiMSum, and Enrich2 (in the development branch of our software github page). Thus, satmut_utils should facilitate uniform processing of MAVE data from several labs using direct variant calling experimental designs.

Moreover, the analysis of CBS is also marred by certain analytical choices as well as the disappointing lack of quantitative agreement between the measurements made by the two library designs in the region in which they overlap. The authors treat the analysis as one of detecting whether library variants are at different frequencies when measuring cDNA vs. genomic DNA. The difficulty introduced by this choice is that the frequency of any particular variant in the cDNA depends on the mRNA levels produced by the other variants in that library and thus does not describe a biological characteristic of the variant. For example, if most variants reduce mRNA levels, a variant that has no effect on mRNA levels will increase in frequency in the cDNA relative to the genomic DNA. The standard solution to deal with this problem is to normalize the change in frequency for each variant by the change in frequency of the wild-type sequence (i.e. calculate the enrichment ratio), which in the

context of the CBS has the interpretation of estimating the mRNA level produced by a variant as a fraction of the wild-type mRNA level, a quantity that can be estimated consistently regardless of library composition. This analysis of enrichment (or sometimes log enrichment) is what is done in the competing DiMSum and Enrich2 packages and what should be done here.

We thank the reviewer for recommending normalization to the wild-type count in derivation of variant effects. We re-analyzed the data with normalization to the wild-type count. Results were largely consistent with our previous analysis, with some variants at high coverage exhibiting increased fitness scores after normalization (**Figure R2**). These variants are predominantly SNPs at high coverage, and their fitness is likely influenced by other errors at the coordinate. We updated all figures impacted by this change: Figures 5-6, Supplementary Figures 5-6.

**Figure R2: Comparison of mRNA abundance estimates for wild-type-normalized and non-normalized frequencies**. For wild-type normalized data, the log ratio between variant count and the inferred wild-type count is plotted (Methods). For non-normalized data, the log ratio between variant count and *total depth* is plotted (inclusion of counts from other variants at the position). In A and B, the Pearson correlation coefficient (r) is shown. **A)** Wild-type normalization impacts log frequency estimates for a small subset of *CBS* variants identified by the amplicon library preparation method. B) Wild-type normalization has a larger impact on the *CBS* log frequency estimates identified by the RACE-like library preparation method (AMP). This is likely due to non-uniform coverage depth across the target, a result of imbalanced bidirectional coverage (positions near primers yield highest depth, especially if fragment lengths are < 100 nt). AMP: Anchored Multiplex PCR (Zheng et al. 2014).

To our knowledge, we are the first to investigate agreement between orthogonal library preparation methods for *direct* variant calling of MAVE data (amplicon vs RACE-like methods). Our prior results comparing the two preparation methods suggested a modest agreement in mRNA abundance effects (Figure 5B). Upon re-analysis (wild-type normalization, filtering variants not observed in all gDNA/cDNA replicates), we found a higher correlation in variant effects albeit for a small number of variants (r=0.998, Supplementary Figure 6B). The small number of shared variants is predominantly attributable to the low coverage of the RACE-like data.

Lower depth of coverage for the RACE-like method is due to uneven uniformity of coverage across each target region. Competition among all *CBS* amplicons is expected with RACE-like adoption of a one-pot PCR reaction to enrich the entire coding sequence. As a result, sequence content and primer efficiency across all fifteen *CBS* amplicons may negatively affect coverage uniformity. For example, coverage of tiles 2 and 4 by the RACE-like method suffered compared to other amplicons, possibly because of high GC content of the amplicons (Figure 5B).

A final comment concerns the applicability of the satmut_utils software to varying MAVE library designs. Specifically, the software is specialized for considering libraries similar to NNS designs but cannot accommodate barcodes or libraries with multiple mutations as would be produced by error prone PCR. This limitation should be made clearer to the reader in the abstract / introduction so that a potential reader will know quickly whether the software described here is able to accommodate their experimental data.

We agree with the reviewer about the importance of clearly specifying these limitations. We have now revised the Introduction and Discussion to clarify these points.

Minor comments:

Page 5 Line 33 "DimSum only annotates standard amino acid changes" The authors should clarify the claim here. DimSum works on nucleotide data as well (e.g. tRNAs), can handle NNS mutagenesis, and can handle multiple amino acid or nucleotide changes in a single read. Similarly pg 9 Line 17, why can't you run DimSum in nucleotide mode to get the nucleotide annotations?

Thanks for pointing out this unclear section in the text. We revised the manuscript to note that DiMSum provides nucleotide strings for each identified variant (nucleotide annotations provided in ‘noncoding’ mode and only for synonymous variants in ‘coding’ mode). To benchmark DiMSum nucleotide-level calls, we would need to write post-processing code to identify the mismatches in the nucleotide string that define the variant. Thus, we decided to compare only amino acid changes for DiMSum to ensure an unbiased analysis.

Page 10 Line 57 "significantly" implies a statistical test. Evidence is strong here, but no test done. Suggest synonym.

Thanks for the suggestion. We edited the text as “... training error correction models on simulated data *appreciably* improves variant calling precision”.

Figure 1C Colored ticks on y axis not explained.

These ticks were included to help the reader track the differences in precision at perfect recall for each variant caller. We decided to remove these tick marks for clarity in the revised manuscript.

Figure 1D The SNP panel does not display the overall distributions of errors very well because all you can see is the high outliers. I suggest showing log (observed/true).

Thanks for the suggestion. We have revised Figure 1D accordingly, and the SNP distribution is now better-visualized.

Figure 5B What is the p-value for the Spearman correlation?

Our results using two recent tRNA measurement approaches indicated a weak correlation between mRNA abundance effect and the tRNA abundance difference between alternate and reference codons. After updates to the analysis (wild-type normalization), these results remained consistent with p-values < 0.1 by a Spearman rank correlation test for both Hydro-tRNAseq and mim-tRNAseq datasets (Figures 6B-D, Supplementary Figure 5C). We now include the p-values in the revised text.

Pg. 18 line 54. The integration with the previous DMS study should be improved. This study provides data capable of estimating defects in mRNA levels of variants relative to the wild-type. Is a substantial mRNA defect sufficient to produce a fitness defect in the yeast complementation assay? How does this impact the interpretation of the previous results on mRNA stability and tRNA abundance?

We thank the reviewer for this suggestion. In the revised manuscript (Supplementary Table 1), we include a list of all differential variants called by both amplicon and RACE-like methods (FDR < 0.15). In this table, we include our results along with several annotations: codon stability coefficient difference, tRNA abundance log ratios, and yeast functional complementation scores.

We also address this concern in a new figure (Figure 6E) and the Discussion section. Our data indicates little correspondence between mRNA abundance effects in human cells and fitness estimates by yeast functional complementation. We propose the following possibilities that may explain this observation.

First, *CBS* activity is differentially-regulated between yeast and humans, as the heme domain is dispensable for activity in yeast, but supports robust activity in human cells, albeit mildly (Maclean et al. 2000; Meier et al. 2001). In our new data, we focused on regions in- and near- the heme domain where the variant effects may be expected to differ most significantly between the two species. Additionally, human CBS, but not yeast CBS, is activated by S-adenosylmethionine binding to the C-terminal domain (Jhee et al. 2000; Maclean et al. 2000). Altogether, several differences exist in the regulation of CBS between humans and yeast, which may partially explain little agreement between readouts.

Second, unpublished results from the Roth laboratory (**Figure R3**) indicate a nonlinear relationship between yeast functional complementation scores and enzyme activity measured in *E. coli* (Kozich et al. 2010), emphasizing the importance of selecting a proper fitness score threshold upon which to consider a variant deleterious. In this analysis, we chose a fitness score cutoff of 0.7 for comparison to mRNA abundance effects, as this approximated ~50% enzyme activity in *E. coli*. However, this decision may not be optimal for comparison of human and yeast MAVE data. Lack of sufficient data from human cells precludes determination of the appropriate fitness score cutoff.

**Figure R3: Relationship between yeast functional complementation fitness and enzyme activity**. Enzyme activity data derive from *CBS* variants expressed in *E. coli* (Kozich et al. 2010) and detected by yeast functional complementation. The Spearman rank correlation coefficient (SRCC) is shown.

Lastly, we found variants with *increased* mRNA abundance that are deleterious at the protein-level: C109G and C109T. These variants increase mRNA abundance in human cells but decrease CBS fitness in yeast. We hypothesize a negative feedback mechanism of tRNA^Cys^ charging on *CBS* mRNA abundance. That is, a defect in *CBS* biosynthesis of cysteine (loss-of-function variants) may negatively feed-back on the *CBS* mRNA by stimulating No-Go Decay at cysteine codons. In support of this hypothesis, we found numerous changes to and from cysteine that alter mRNA (detailed in Supplementary Table 1). We note changes involving serine and methionine may also be subject to feedback regulation, as these amino acids might increase in concentration under null CBS variants.

Altogether, differences between human and yeast model systems (heme, SAM activation); the nonlinear relationship between functional complementation scores and enzyme activity; and biosynthetic feedback mechanisms may partly explain a poor correspondence between *CBS* variant effects on mRNA abundance in human cells and in the yeast complementation assay.

**Second round of review**

**Reviewer 1:** In the new version of the manuscript, you addressed all the point I have previously raised. I suggested the Editor to accept your manuscript for publication.
